# C-reactive protein–triglyceride glucose index for the detection of the severity of coronary artery disease in different glucose metabolic states in patients with H-type hypertension

**DOI:** 10.1186/s12933-026-03318-6

**Published:** 2026-08-09

**Authors:** Li Luo, Lian Wang, Xian Yu, Qiuyue Yang, Xian Ming, Hongbo Xiong, Dongsheng Li, Xisheng Yan

**Affiliations:** 1 School of Medicine, Wuhan University of Science and Technology, Wuhan, 430060 Hubei China; 2 Department of Cardiology, Wuhan Third Hospital & Tongren Hospital of Wuhan University, Wuhan, 430074 Hubei China; 3 Department of Cardiology, Suizhou Hospital, Hubei University of Medicine, Suizhou, 441300 Hubei China

**Keywords:** C-reactive protein–triglyceride glucose index, Coronary artery disease, H-type hypertension, Glucose metabolism status

## Abstract

**Background:**

The C-reactive protein–triglyceride glucose index (CTI) is a novel composite biomarker that integrates inflammation and insulin resistance. However, its association with the severity of coronary artery disease (CAD) in patients with H-type hypertension under different glucose metabolism statuses remains unclear.

**Methods:**

This retrospective study enrolled 1392 hypertensive patients who underwent coronary angiography. CTI was calculated as 0.412 × ln(CRP[mg/L]) + ln(TG[mg/dL] × FPG [mg/dL]/2). Patients were divided into three groups according to CTI tertiles: T1 group (6.748 ≤ CTI < 8.674), T2 group (8.674 ≤ CTI < 9.549), and T3 group (9.549 ≤ CTI ≤ 12.713). Based on the Gensini score derived from coronary angiography, the study cohort was divided into three groups according to the severity of stenosis: low stenosis, medium stenosis, and high stenosis. Logistic regression, restricted cubic spline (RCS) analysis, and receiver operating characteristic (ROC) curve analysis were used to investigate the association between CTI and CAD severity across different glucose metabolism statuses and H‑type hypertension subgroups. Incremental utility of CTI was evaluated using receiver operating characteristic (ROC) curves, net reclassification improvement (NRI), integrated discrimination improvement (IDI), and decision curve analysis (DCA).

**Results:**

The results show that CTI is significantly associated with CAD severity in hypertensive patients. After adjusting for risk factors, CTI remained significantly associated with multi-vessel CAD (OR = 2.155, 95%CI 1.839–2.455) and moderate-to-severe coronary stenosis (OR = 1.818, 95% CI 1.603–2.062). RCS analysis revealed a linear dose–response relationship between CTI and CAD severity (P for non-linearity > 0.05). Notably, we observed significant interaction between CTI and H-type hypertension on CAD severity (*P* = 0.033). Under different glycemic statuses, high CTI levels were associated with CAD severity; however, no association between CTI and moderate‑to‑severe coronary stenosis was observed in patients with diabetes mellitus (DM). In the DM subgroup of patients with H-type hypertension, after adjustment for risk factors, patients in the T3 group had a 6.475-fold increased risk of multi-vessel CAD compared with those in the T1 group (95% CI 1.693–24.773, *P* = 0.006). ROC analysis demonstrated that, across different glucose metabolism subgroups, CTI showed the highest discriminative ability for multi‑vessel CAD in patients with H‑type hypertension and comorbid DM (AUC = 0.712, 95% CI 0.619–0.804, *P* < 0.001).

**Conclusion:**

CTI is an independently associated factor for coronary stenosis severity in patients with hypertension, particularly in those with H‑type hypertension and comorbid diabetes. This simple and cost‑effective composite biomarker holds promise as a practical tool for early risk stratification and for guiding individualized prevention strategies.

**Supplementary Information:**

The online version contains supplementary material available at https://doi.org/10.1186/s12933-026-03318-6.

## Introduction

Cardiovascular disease (CVD) remains the leading cause of death worldwide. Coronary heart disease (CHD) is the major component of CVD and, together with stroke, accounts for nearly 85% of all global CVD deaths. Although the age-standardized mortality rate of CHD has shown a downward trend over the past three decades, the absolute number of CHD cases, deaths, and disability burden has continued to rise due to population growth and aging [1], placing enormous pressure on healthcare systems globally.

H-type hypertension, characterized by the coexistence of hypertension and hyperhomocysteinemia (Hcy), has increasingly been recognized as a distinct inflammatory–metabolic phenotype rather than merely a subtype of hypertension [2]. H-type hypertension is generally defined as hypertension accompanied by plasma Hcy concentrations > 10 μmol/L [3, 4]; however, hcy levels ≥ 15 µmol/L have been reported as a cutoff value [5–7]. Accounts for approximately 80% of patients with essential hypertension. This patient population has a significantly higher risk of cardiovascular events, with an approximately fivefold increase compared with patients with essential hypertension alone, and a 25- to 30-fold increase compared with the general population [3]. Hcy is a sulfur‑containing non‑essential amino acid derived as an intermediate metabolite of methionine. In patients with hyperhomocysteinemia, methionine metabolism is impaired due to genetic defects in genes encoding metabolic enzymes or deficiencies in specific vitamin cofactors, leading to elevated Hcy levels [8]. Hcy levels are associated with insulin resistance and the risk of death in patients with diabetes [3]. Elevated Hcy levels are closely associated with the severity of coronary artery stenosis and may serve as an adjunctive biomarker beyond traditional risk factors [9, 10]. Compared with conventional hypertension, H-type hypertension is characterized by enhanced endothelial dysfunction, oxidative stress, chronic vascular inflammation, and accelerated atherosclerosis, resulting in a substantially greater burden of cardiovascular disease [11]. Hcy, the defining biochemical feature of H-type hypertension, may interact with inflammatory and metabolic pathways, thereby amplifying vascular injury beyond the effect of blood pressure elevation alone [11, 12].

Diabetes may increase cardiovascular risk by accelerating the progression of atherosclerosis [13]. Multiple studies have shown that among patients with coronary heart disease under different glucose metabolism statuses, the severity of coronary artery stenosis is greater in the diabetes mellitus (DM) group than in the normal glucose regulation (NGR) group [14]. Furthermore, the plaque burden was also most pronounced in the DM group [15, 16].Insulin resistance (IR) refers to the impaired biological action of insulin in target tissues, manifested as abnormal receptor function and reduced sensitivity, and is closely associated with hypertension, diabetes mellitus, and dyslipidemia [17–19]. IR is also a marker of systemic inflammation, endothelial dysfunction, oxidative stress, and a prothrombotic state. It can induce endothelial damage and atherosclerosis, thereby promoting the development and progression of atherosclerotic cardiovascular diseases, including coronary artery disease [20–22]. Therefore, IR is regarded as an important mediator of cardiovascular events and a reliable biomarker for predicting cardiovascular disease [23].

In recent years, the triglyceride-glucose (TyG) index has emerged as a reliable surrogate marker of insulin resistance and dyslipidemia that has garnered considerable attention, owing to its ease of calculation, low cost, and strong association with the risk of cardiovascular events [24, 25]. Previous studies have evaluated the predictive value of the TyG index for adverse outcomes in patients with various cardiovascular disease phenotypes [26–28]. However, these studies have only emphasized the exacerbating role of insulin resistance in the progression of cardiovascular disease, while neglecting the possibility that systemic inflammation may participate in this process through synergistic or mediating effects. There is a bidirectional interaction between inflammation and IR [29]. Inflammation is considered a key component in the pathogenesis of coronary heart disease. The inflammatory response can significantly increase the risk of coronary heart disease events by promoting atherosclerosis, impairing endothelial function, and facilitating thrombosis [30]. C-reactive protein (CRP) is a classic non‑specific inflammatory biomarker. It is an IL‑6‑induced hepatic acute‑phase reactant that is closely associated with coronary heart disease and has been widely recognized as a reliable cardiovascular marker of systemic inflammation [31, 32]. However, relying on a single biomarker alone may not fully capture the complex interplay between metabolic dysfunction and inflammatory processes. The composite C-reactive protein–triglyceride–glucose index (CTI) developed by Ruan et al. [33] simultaneously reflects both insulin resistance (IR) and inflammatory status, thereby enabling a more comprehensive assessment of cardiovascular risk. CTI is associated with metabolic syndrome, cardiometabolic risk, and all-cause mortality [34]. CTI has been demonstrated to effectively predict all‑cause mortality and cardiovascular mortality in patients with coronary heart disease and comorbid type 2 diabetes [35].

Although CTI has been extensively associated with CAD severity and adverse cardiovascular outcomes, previous studies have largely evaluated CTI in heterogeneous populations and have rarely examined whether its clinical significance varies according to inflammatory–metabolic phenotypes [36, 37]. Therefore, whether H-type hypertension modifies the association between CTI and CAD severity remain important unanswered questions. Accordingly, this study was designed to investigate whether the inflammatory–metabolic phenotype, as exemplified by H type hypertension, modifies the association between CTI and angiographic CAD severity, as well as the influence of different glucose metabolic states on this relationship.

## Methods

### Study design and population

The data for this study were derived from hypertensive patients admitted to the Department of Cardiology at Wuhan Third Hospital between January 2021 and March 2024 who underwent coronary angiography. The inclusion criteria of this study were as follows: (1) the diagnostic criteria for hypertension included a history of hypertension, current use of antihypertensive medication, systolic blood pressure (SBP) ≥ 140 mmHg, or diastolic blood pressure (DBP) ≥ 90 mmHg; (2) subjects with at least one major coronary artery stenosis ≥ 50% were diagnosed with coronary artery disease (CAD); (3) all participants underwent coronary angiography according to the study protocol. The exclusion criteria were as follows: (1) patients with incomplete clinical data or coronary angiography results; (2) patients aged < 18 years, or pregnant women; (3) patients with severe comorbidities, including advanced cancer, end-stage liver or kidney failure, or significant hematological/endocrine diseases; (4) patients with mental or cognitive impairments; (5) patients with < 50% stenosis of the main lumen in the coronary angiography report; (6) patients who had recently taken folic acid or B vitamins.

**Fig. 1:**
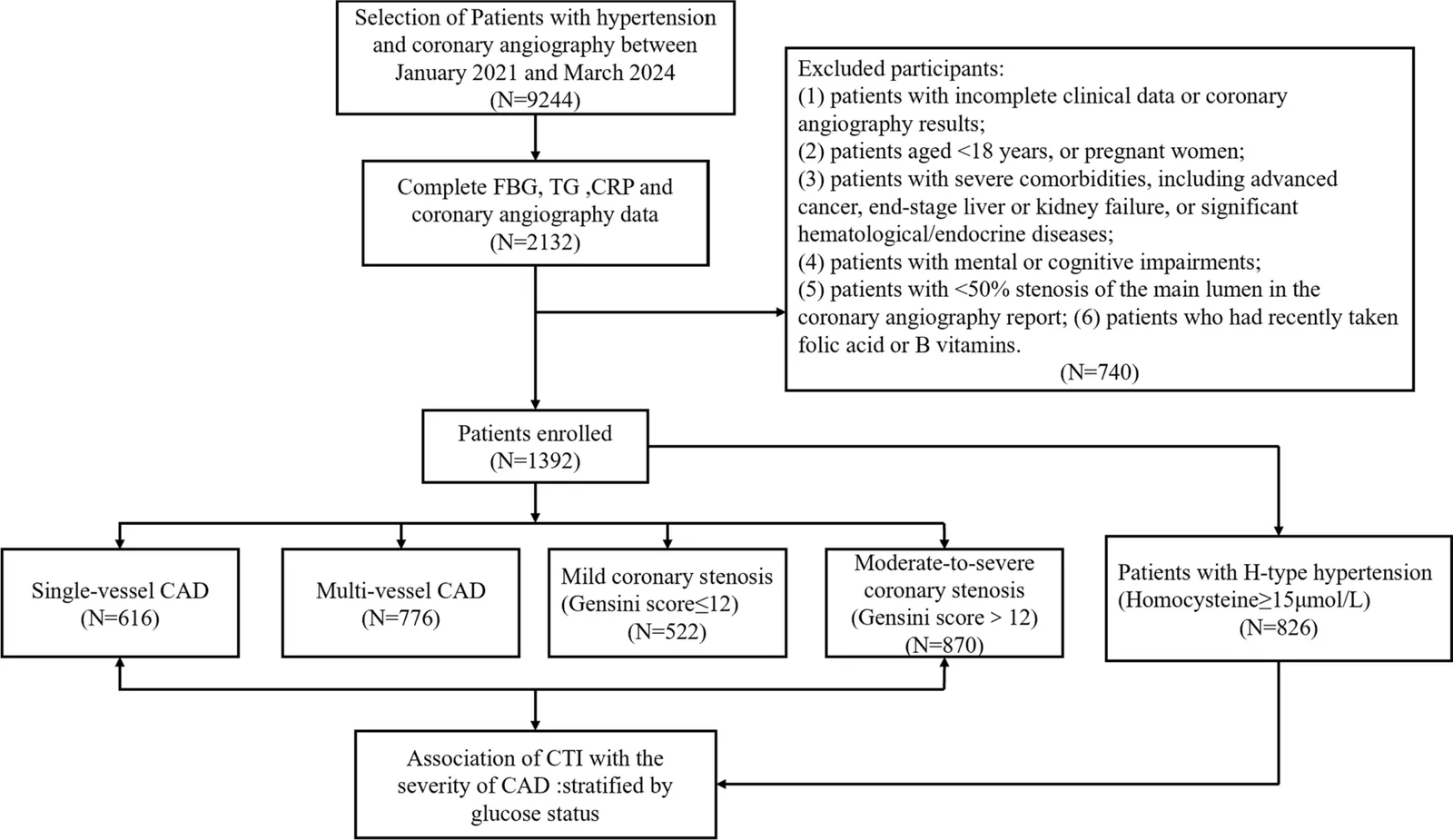
Flow chart of the study design. This figure illustrates the process of participant selection for the retrospective cohort study

The overall study design and analytical framework are shown in Fig. 1. Finally, after screening according to the pre‑specified exclusion criteria, a total of 1,392 subjects were enrolled. Based on the diagnostic criteria established by the American Heart Association [38], the enrolled patients were divided into a single‑vessel coronary artery disease group (N = 616) and a multi‑vessel coronary artery disease group (N = 776). The study protocol was approved by the Ethics Committee of Wuhan Third Hospital (approval number: KY2026-025), complied with the requirements of the Declaration of Helsinki, and written informed consent was obtained from all participants.

### Data collection and definitions

Demographic and clinical characteristics, including age, sex, height, weight, smoking history, drinking history, and medical history, were collected through the electronic medical record system. Blood samples were collected by trained clinical personnel after an overnight fast of at least 8 h on the morning of the day following hospital admission. The measured parameters included fasting plasma glucose (FPG), glycated hemoglobin (HbA1c), total cholesterol (TC), triglycerides (TG), high‑density lipoprotein cholesterol (HDL‑C), low‑density lipoprotein cholesterol (LDL‑C), homocysteine, uric acid (UA), alanine aminotransferase (ALT), aspartate aminotransferase (AST), C‑reactive protein (CRP), red blood cell count (RBC), white blood cell count (WBC), neutrophil count (NEUT), lymphocyte count, and platelet count. After five minutes of rest, blood pressure was measured three times, and the average value was used for analysis.

In this study, DM was defined based on any of the following criteria: (1) a previous established diagnosis of diabetes with complete medical history records; (2) current use of any glucose-lowering therapy (including oral antidiabetic agents and insulin); or (3) meeting the diagnostic criteria of the 2019 European Society of Cardiology (ESC) and the European Association for the Study of Diabetes (EASD) guidelines [39], fasting plasma glucose (FPG) ≥ 7.0 mmol/L or HbA1c ≥ 6.5%. According to the criteria of the 2019 guidelines on diabetes, pre-diabetes, and cardiovascular diseases developed jointly by the ESC/EASD, the study subjects were divided into three groups [39]: normal glucose regulation (NGR) was defined as FPG < 5.6 mmol/L or HbA1c < 5.7%; pre-diabetes (Pre-DM) was defined as FPG 5.6–6.9 mmol/L or HbA1c 5.7–6.4%; and diabetes mellitus (DM) was defined as FPG ≥ 7.0 mmol/L or HbA1c ≥ 6.5%. In the present study, H-type hypertension was operationally defined as hypertension accompanied by plasma homocysteine levels ≥ 15 μmol/L for risk stratification purposes; The CTI was calculated using the formula: CTI = 0.412 × ln(CRP [mg/L]) + ln(TG [mg/dL] × FPG[mg/dL]/2) [33]; The TyG was calculated using the formula: TyG = Ln[TG (mg/dL) × FPG(mg/dL)]/2 [40]; The METS-IR was calculated using the formula: METS-IR = (Ln [(2 × FPG) + TG] × BMI)/Ln[HDL-C]) [41].

Coronary angiography data were interpreted and recorded by cardiologists. The diagnostic criterion for CAD was the presence of ≥ 50% luminal stenosis in at least one major coronary artery, including the left anterior descending artery, left circumflex artery, or right coronary artery, on coronary angiography. Coronary artery disease severity was assessed according to the number of involved vessels, and CHD was further classified as single‑vessel disease involving only one major coronary artery or multi‑vessel disease involving two or more major coronary arteries [42]. Gensini scores were calculated by experienced cardiologists based on patients’ coronary angiography results to quantify the overall severity of coronary artery stenosis. According to tertiles of the Gensini score, the lowest tertile ≤ 12 points was defined as mild coronary stenosis, and the middle and highest tertiles > 12 points were combined and defined as moderate‑to‑severe coronary stenosis. This variable was used as a binary outcome in some analyses. Multivessel disease was uniformly defined as an anatomical indicator reflecting the extent of coronary involvement, as determined by the number of diseased vessels; moderate-to-severe coronary stenosis was defined based on the Gensini score, which represents the overall burden and severity of coronary atherosclerosis; and the term “severity of coronary artery disease” was used solely as a general descriptor encompassing these two complementary indicators.

### Statistical analysis

Normally distributed variables are presented as mean ± standard deviation, and non‑normally distributed variables are presented as median (25th to 75th percentiles). Categorical variables are expressed in frequency and percentage. Comparisons of continuous variables were performed using independent samples t‑test for normally distributed data or Mann‑Whitney U test for non‑normally distributed data; categorical variables were compared using the chi‑square test. To control for multiple comparisons, the Benjamini–Hochberg procedure was applied to control the false discovery rate [43], and adjusted *P*-values were reported where appropriate. The detailed adjusted P-values are provided in Supplementary Table S11.

The Least Absolute Shrinkage and Selection Operator (LASSO) regression was used to reduce dimensionality and select potential variables. The λ value that minimized the mean square error was chosen through tenfold cross‑validation, and variables with non‑zero coefficients were ultimately retained. To investigate potential collinearity between the CTI and other covariates, variance inflation factor (VIF) was examined. A multivariate logistic regression model was then constructed based on the variables selected by LASSO. The SHAP (Shapley Additive exPlanations) method was used to interpret the model output and to evaluate the contribution of each feature to the outcome. The SHAP beeswarm plot displays the relationship between feature values (red‑blue gradient) and SHAP values, and the mean absolute SHAP value is used to rank variable importance. Additionally, SHAP dependence curves were plotted to verify the direction of the association between each key variable and the outcome. Based on the final multivariate logistic regression model, a nomogram was constructed to visually evaluate the association of each variable coronary artery stenosis.

Patients were divided into three groups according to CTI tertiles: T1 group (6.748 ≤ CTI < 8.674), T2 group (8.674 ≤ CTI < 9.549), and T3 group (9.549 ≤ CTI ≤ 12.713). In addition, patients were classified into three groups based on Gensini score tertiles: lowest tertile (≤ 12), mid tertile (12–35), and highest tertile (≥ 35). To enhance the robustness of the study findings, the Gensini score was also evaluated as a continuous variable. Logistic regression was used to analyze the association between CTI and the severity of CAD, including single‑vessel versus multi‑vessel disease and mild versus moderate‑to‑severe stenosis, and to calculate odds ratios (OR) with their 95% confidence intervals (CI). Patients were classified by Hcy (< 15 μmol/L, ≥ 15 μmol/L) and CTI tertiles (T1, T2, T3), yielding six combined exposure groups. Using CTI T1 with Hcy < 15 μmol/L as reference, multivariate logistic regression was performed to assess the joint associations with coronary stenosis. Restricted cubic spline (RCS) regression was used to evaluate the dose‑response relationship between CTI and CAD severity. Four knots were placed at the 5th, 35th, 65th, and 95th percentiles. The non‑linear model was compared with the linear model using the likelihood ratio test, and the non‑linear trend was determined by the *P* value for non‑linearity, with *P* < 0.05 indicating significant non‑linearity. Receiver operating characteristic (ROC) curves were plotted, and the area under the curve (AUC) was calculated to assess the discriminative ability of CTI and other indices (TyG, FPG, METS‑IR) for the severity of CAD. The optimal cut‑off value was determined using the Youden index, and the corresponding sensitivity and specificity were reported. In addition, the Net Reclassification Improvement (NRI) and Integrated Discrimination Improvement (IDI) were calculated to further evaluate the incremental discriminative value of CTI beyond the conventional metabolic indices (TyG, FPG, METS-IR). The 95% confidence intervals and *P* values for NRI and IDI were derived from the bootstrap method (1000 resamples), while the AUC improvements were tested using the DeLong method. Hypertensive patients were divided into groups according to glucose metabolic statuses: NGR, Pre‑DM, and DM. In each subgroup, CTI was treated as both a continuous variable and a categorical variable in univariate and multivariate binary logistic regression analyses to calculate ORs and their 95% CIs. At the same time, RCS was used to evaluate the dose‑response relationship, and ROC curves were plotted to calculate the AUC. From all hypertensive patients, those with Hcy ≥ 15 μmol/L were selected as the H‑type hypertension subgroup and the above‑mentioned logistic regression, RCS, and ROC analyses stratified by glucose metabolic statuses were repeated.

Subgroup analyses were performed according to sex, age (< 60 years, ≥ 60 years), BMI (< 24, 24–28, ≥ 28 kg/m^2^), smoking, drinking, hyperuricemia, and NAFLD. Multivariate logistic regression was repeated in each subgroup, and interaction terms were introduced to test for effect modification, with the interaction effects calculated. Calibration curves were used to assess the agreement between estimated probabilities and observed probabilities, and the bias‑corrected goodness‑of‑fit was calculated using bootstrap resampling (1000 replicates). Decision curve analysis (DCA) was used to evaluate the clinical net benefit of the CTI-based risk model at different thresholds and to compare it with the “treat all” or “treat none” strategies.

All data were analyzed using IBM SPSS Statistics version 24.0 (Chicago, IL, USA), GraphPad Prism 9.5.1 (SanDiego, California, USA), and R version 4.4.1 (R Foundation for Statistical Computing, Vienna, Austria). The visualization of the results was carried out using the ggplot2 and rms packages within the R program. All statistical tests were two‑sided, and a *P* value < 0.05 was considered statistically significant.

## Results

###  Study population baseline characteristics

A total of 1392 hypertensive patients who underwent coronary angiography were enrolled in this study. The median age was 68.0 years (IQR 61–75), and 740 (53.2%) were male. Among them, 776 patients had multi‑vessel disease, and 870 patients had moderate‑to‑severe coronary stenosis.

Patients were divided into three groups according to CTI tertiles: T1 (6.74 ≤ CTI ≤ 8.66), T2 (8.67 ≤ CTI ≤ 9.54), and T3 (9.55 ≤ CTI ≤ 12.72), as detailed in Table 1. Compared with patients in the lowest CTI tertile group (T1), those in the highest tertile group (T3) had higher levels of BMI, FPG, HbA1c, TC, TG, LDL‑C, UA, WBC, NEUT, CRP, and platelets, as well as lower levels of HDL‑C and lymphocytes (*P* < 0.05). There were no significant differences in age, height, weight, systolic blood pressure, diastolic blood pressure, smoking, drinking, hemoglobin, or AST among the groups (*P* > 0.05). Regarding medication use, only statins showed a significant difference among the groups (*P* = 0.046). Both Gensini score and the proportion of multi‑vessel disease increased with rising CTI levels (both *P* < 0.001). Compared with the single‑vessel disease group, the multi‑vessel disease group had higher proportions of male patients, prevalence of H‑type hypertension, and prevalence of abnormal glucose metabolism (all *P* < 0.05), as detailed in Table 2.

**Table 1:** Baseline characteristics according to CTI tertiles

Characteristics	Tertiles of CTI			*P* value
	T1(6.748–8.674) (n = 464)	T2(8.674–9.549) (n = 464)	T3(9.549–12.713) (n = 464)	
Sex, n (%)				0.047
Female	221 (47.6%)	234 (50.4%)	197 (42.5%)	
Male	243 (52.4%)	230 (49.6%)	267 (57.5%)	
Age, years	67.00 (60.25, 74.00)	66.00 (60.00, 75.00)	69.00 (61.00, 75.00)	0.081
Height, m	1.65 (1.60, 1.70)	1.65 (1.60, 1.70)	1.65 (1.60, 1.70)	0.880
Weight, kg	65.00 (60.00, 72.88)	66.00 (60.00, 75.00)	67.00 (60.00, 75.00)	0.135
BMI, kg/m^2^	24.09 (22.49, 25.95)	24.44 (22.86, 26.57)	24.85 (23.20, 26.82)	< 0.001
SBP, mmHg	142 (130.00, 157.75)	145.00 (132.00, 160.00)	146.00 (132.00, 159.00)	0.159
DBP, mmHg	83.00 (75.00, 91.00)	84.00 (78.00, 92.75)	84.00 (75.00, 91.00)	0.128
Smoking, n (%)	141 (30.4%)	136 (29.3%)	164 (35.3%)	0.109
Drinking, n (%)	52 (11.2%)	37 (8.0%)	45 (9.7%)	0.248
Hyperuricemia, n (%)	50 (10.8%)	74 (15.9%)	84 (18.1%)	0.006
NAFLD, n (%)	27 (5.8%)	55 (11.9%)	69 (14.9%)	< 0.001
H-type hypertension, n (%)	240 (51.7%)	272 (58.6%)	314 (67.7%)	< 0.001
*Laboratory measurements*				
RBC (× 10^12^/L)	4.41 (4.09, 4.76)	4.46 (4.12, 4.85)	4.52 (4.19, 4.88)	0.009
WBC (× 10^9^/L)	5.70 (4.66, 6.80)	6.16 (5.20, 7.41)	6.68 (5.70, 8.28)	< 0.001
Homocysteine (μmol/L)	15.28 (12.36, 19.05)	16.09 (12.89, 20.59)	17.45 (13.77, 22.39)	< 0.001
Hb (g/L)	133.00 (123.00, 143.00)	134.00 (124.00, 146.00)	136.00 (124.00, 147.75)	0.066
NEUT (× 10^9^/L)	62.30 (56.05, 68.80)	64.20 (58.02, 70.60)	67.00 (60.70, 72.48)	< 0.001
Lymphocyte (× 10^9^/L)	28.80 (22.50, 34.90)	27.25 (21.50, 32.70)	24.80 (19.50, 30.30)	< 0.001
CRP (mg/L)	0.40 (0.20, 0.70)	1.10 (0.50, 2.26)	2.70 (1.40, 6.85)	< 0.001
Platelet (× 10^9^/L)	206.50 (168.00, 235.80)	210.10 (178.63, 250.00)	220.50 (181.50, 260.00)	< 0.001
FPG (mmol/L)	5.46 (4.92, 6.41)	6.26 (5.27, 7.65)	8.43 (6.60, 11.64)	< 0.001
HbA1c (%)	5.95 (5.50, 6.50)	6.00 (5.60, 6.70)	6.45 (5.80, 7.50)	< 0.001
TC (mmol/L)	4.31 (3.41, 5.02)	4.47 (3.77, 5.18)	4.74 (3.87, 5.61)	< 0.001
TG (mmol/L)	1.06 (0.82, 1.41)	1.70 (1.26, 2.23)	2.58 (1.88, 3.74)	< 0.001
LDL-C (mmol/L)	2.24 (1.64, 2.84)	2.49 (1.96, 3.00)	2.58 (2.00, 3.24)	< 0.001
HDL-C (mmol/L)	1.35 (1.14, 1.62)	1.19 (1.01, 1.41)	1.07 (0.90, 1.27)	< 0.001
ALT (U/L)	18.00 (14.00, 24.75)	19.00 (14.00, 28.00)	21.00 (15.00, 33.00)	< 0.001
AST (U/L)	21.00 (18.00, 26.00)	21.00 (17.00, 26.00)	21.00 (17.00, 30.75)	0.242
UA (μmol/L)	335.50 (271.25, 402.00)	369.00 (308.25, 442.00)	389.50 (331.00, 484.75)	< 0.001
*Medications, n (%)*				
CCB	178 (38.4%)	192 (41.4%)	202 (43.5%)	0.274
Statin	42 (9.1%)	23 (5.0%)	37 (8.0%)	0.046
Antiplatelet	46 (9.9%)	36 (7.8%)	45 (9.7%)	0.455
Beta blockers	71 (15.3%)	57 (12.3%)	58 (12.5%)	0.321
ACEI/ARB	112 (24.1%)	105 (22.6%)	107 (23.1%)	0.855
Glucose metabolism state, n (%)				< 0.001
NGR	316 (68.1%)	221 (47.6%)	118 (25.4%)	
Pre-DM	120 (25.9%)	166 (35.8%)	152 (32.8%)	
DM	28 (6.0%)	77 (16.6%)	194 (41.8%)	
CTI	8.20 (7.84, 8.46)	9.13 (8.91, 9.32)	10.20 (9.88, 10.69)	< 0.001
Gensini score	12.00 (6.00, 24.00)	24.00 (9.00, 38.00)	33.00 (15.00, 46.00)	< 0.001
TyG index	8.50 (8.18, 8.78)	9.11 (8.75, 9.38)	9.80 (9.41, 10.24)	< 0.001
METS-IR index	20.91 (19.07, 22.78)	22.25 (20.30, 24.39)	23.75 (21.18, 25.92)	< 0.001
Number of vessels with stenosis				< 0.001
Single-vessel CAD	308 (66.4%)	195 (42.0%)	113 (24.4%)	
Multi-vessel CAD	156 (33.6%)	269 (58.0%)	351 (75.6%)	

Body mass index (BMI), Systolic blood pressure (SBP), Diastolic blood pressure (DBP), Non-alcoholic fatty liver disease (NAFLD), Red blood cell (RBC), White blood cell (WBC), Hemoglobin (Hb), Neutrophil (NEUT), C-reactive protein (CRP), Fasting plasma glucose (FPG), Glycated hemoglobin A1c (HbA1c), Total cholesterol (TC), Triglyceride (TG), Low-density lipoprotein cholesterol (LDL-C), High-density lipoprotein cholesterol (HDL-C), Alanine aminotransferase (ALT), Aspartate aminotransferase (AST), Uric acid (UA), Calcium channel blocker (CCB), Angiotensin-converting enzyme inhibitor (ACEI), Angiotensin Ⅱ receptor blocker (ARB), Normal glucose regulation (NGR), Pre-diabetes mellitus (Pre-DM), Diabetes mellitus (DM), C-reactive protein-triglyceride-glucose index (CTI), Triglyceride-glucose index (TyG), Metabolic score for insulin resistance (METS-IR), Coronary artery disease (CAD)

**Table 2:** Baseline characteristics according to severity of CAD

characteristics	Total (n = 1392)	Single-vessel CAD (n = 616)	Multi-vessel CAD (n = 776)	*P* value
Sex, n(%)				< 0.001
Female	652 (46.8%)	327 (53.1%)	325 (41.9%)	
Male	740 (53.2%)	289 (46.9%)	451 (58.1%)	
Age, years	68.00 (61.00, 75.00)	68.00 (61.00, 74.00)	69.00 (61.00, 75.00)	0.420
Height, m	1.65 (1.60, 1.70)	1.63 (1.59, 1.70)	1.66 (1.60, 1.71)	0.001
Weight, kg	66.00 (60.00, 74.00)	65.00 (60.00, 72.00)	68.00 (60.00, 75.00)	< 0.001
BMI, kg/m^2^	24.42 (22.84, 26.42)	23.88 (22.22, 25.63)	24.97 (23.33, 27.05)	< 0.001
SBP, mmHg	144 (131.00, 158.00)	144.00 (132.00, 157.00)	145.00 (130.00, 160.00)	0.653
DBP, mmHg	84.00 (75.00, 92.00)	84.00 (77.00, 91.00)	84.00 (75.00, 92.00)	0.434
Smoking, n (%)	441 (31.7%)	166 (26.9%)	275 (35.4%)	0.001
Drinking, n (%)	134 (9.6%)	58 (9.4%)	76 (9.8%)	0.812
Hyperuricemia, n (%)	208 (14.9%)	83 (13.5%)	125 (16.1%)	0.171
NAFLD, n (%)	151 (10.8%)	49 (8.0%)	102 (13.1%)	0.002
H-type hypertension, n (%)	826 (59.3%)	342 (55.5%)	484 (62.4%)	0.010
*Laboratory measurements*				
RBC (× 10^12^/L)	4.45 (4.12, 4.82)	4.44 (4.09, 4.77)	4.48 (4.14, 4.87)	0.019
WBC (× 10^9^/L)	6.20 (5.12, 7.50)	5.86 (4.87, 7.00)	6.47 (5.47, 7.86)	< 0.001
Homocysteine (μmol/L)	16.20 (12.97, 20.59)	15.62 (12.59, 19.33)	16.74 (13.20, 21.48)	< 0.001
Hb (g/L)	134.00 (123.00,146.00)	134.00 (123.00, 144.00)	134.00 (124.00, 146.75)	0.478
NEUT (× 10^9^/L)	64.65 (58.10, 70.75)	63.50 (57.20, 69.70)	65.40 (59.05, 72.30)	< 0.001
Lymphocyte (× 10^9^/L)	27.00 (20.80, 32.78)	27.70 (22.20, 33.60)	26.25 (19.90, 32.08)	< 0.001
CRP (mg/L)	1.04 (0.40, 2.60)	0.70 (0.20, 1.80)	1.38 (0.50, 3.29)	< 0.001
Platelet(× 10^9^/L)	211.00 (175.00, 249.85)	209.00 (171.00,245.00)	213.00 (178.00,255.00)	0.015
FPG(mmol/L)	6.42 (5.28, 8.37)	5.80 (5.06, 6.91)	7.19 (5.60, 9.77)	< 0.001
HbA1c (%)	6.10 (5.60, 6.90)	6.00 (5.50, 6.50)	6.30 (5.60, 7.20)	< 0.001
TC (mmol/L)	4.50( 3.70, 5.27)	4.41 (3.57, 5.10)	4.59 (3.76, 5.40)	0.002
TG (mmol/L)	1.64 (1.07, 2.45)	1.40 (0.96, 2.06)	1.90 (1.24, 2.70)	< 0.001
LDL-C (mmol/L)	2.44 (1.84, 3.03)	2.31 (1.77, 2.90)	2.49 (1.91, 3.17)	< 0.001
HDL-C (mmol/L)	1.20 (1.00, 1.45)	1.26 (1.06, 1.53)	1.16 (0.97, 1.38)	< 0.001
ALT (U/L)	20.00 (14.00, 29.00)	19.00 (14.00, 26.00)	20.00 (15.00, 31.00)	0.010
AST (U/L)	21.00 (18.00, 27.00)	21.00 (18.00, 26.00)	21.00 (17.25, 28.00)	0.550
UA (μmol/L)	363.00 (301.00, 442.00)	348.50 (286.25, 428.75)	373.00 (316.25, 447.00)	< 0.001
*Medications, n (%)*				
CCB	572 (41.1%)	246 (39.9%)	326 (42.0%)	0.434
Statin	102 (7.3%)	45 (7.3%)	57 (7.3%)	0.977
Antiplatelet	127 (9.1%)	59 (9.6%)	68 (8.8%)	0.600
Beta blockers	186 (13.4%)	88 (14.3%)	98 (12.6%)	0.367
ACEI/ARB	324 (23.3%)	141 (22.9%)	183 (23.6%)	0.761
Glucose metabolism state, n (%)				< 0.001
NGR	655 (47.1%)	350 (56.8%)	305 (39.3%)	
Pre-DM	438 (31.5%)	210 (34.1%)	228 (29.4%)	
DM	299 (21.5%)	56 (9.1%)	243 (31.3%)	
CTI	9.13 (8.46, 9.88)	8.68 (8.16, 9.35)	9.44 (8.83, 10.17)	< 0.001
Gensini score	21.50 (8.00, 38.00)	7.00 (5.00, 12.00)	36.00 (27.00, 47.00)	< 0.001
TYG	9.09 (8.59, 9.63)	8.78 (8.37, 9.29)	9.29 (8.84, 9.83)	< 0.001
METS-IR	22.09 (20.03, 24.53)	21.27 (19.21, 23.10)	22.98 (20.72, 25.28)	< 0.001

Body mass index (BMI), Systolic blood pressure (SBP), Diastolic blood pressure (DBP), Non-alcoholic fatty liver disease (NAFLD), Red blood cell (RBC), White blood cell (WBC), Hemoglobin (Hb), Neutrophil (NEUT), C-reactive protein (CRP), Fasting plasma glucose (FPG), Glycated hemoglobin A1c (HbA1c), Total cholesterol (TC), Triglyceride (TG), Low-density lipoprotein cholesterol (LDL-C), High-density lipoprotein cholesterol (HDL-C), Alanine aminotransferase (ALT), Aspartate aminotransferase (AST), Uric acid (UA), Calcium channel blocker (CCB), Angiotensin-converting enzyme inhibitor (ACEI), Angiotensin Ⅱ receptor blocker (ARB), Normal glucose regulation (NGR), Pre-diabetes mellitus (Pre-DM), Diabetes mellitus (DM), C-reactive protein-triglyceride-glucose index (CTI), Triglyceride-glucose index (TyG), Metabolic score for insulin resistance (METS-IR)

Patients were divided according to Gensini score tertiles: lowest tertile (Gensini score ≤ 12), middle tertile (12 < Gensini score < 35), and highest tertile (Gensini score ≥ 35). The lowest tertile was defined as the mild stenosis group (n = 522), and the middle and highest tertiles were combined as the moderate‑to‑severe stenosis group (n = 870), as detailed in Table S1. Patients in the highest Gensini tertile group had higher levels of male proportion, BMI, WBC, homocysteine, NEUT, CRP, FPG, HbA1c, TC, TG, UA, CTI, TyG, and METS‑IR; higher prevalence of H‑type hypertension, diabetes, and NAFLD; and higher frequency of smoking. No significant differences in medication history were observed among the groups (all *P* > 0.05). Notably, the proportion of multi‑vessel CAD in the highest Gensini tertile was significantly higher than that in the lowest tertile (*P* < 0.001). Regardless of whether assessing multi‑vessel disease or moderate‑to‑severe stenosis, CTI, TyG index, METS‑IR index, CRP, FPG, HbA1c, LDL‑C, and TG all showed significant positive correlations, whereas HDL‑C showed a negative correlation (all *P* < 0.05).

### Identification of factors associated with the severity of coronary artery stenosis

**Fig. 2:**
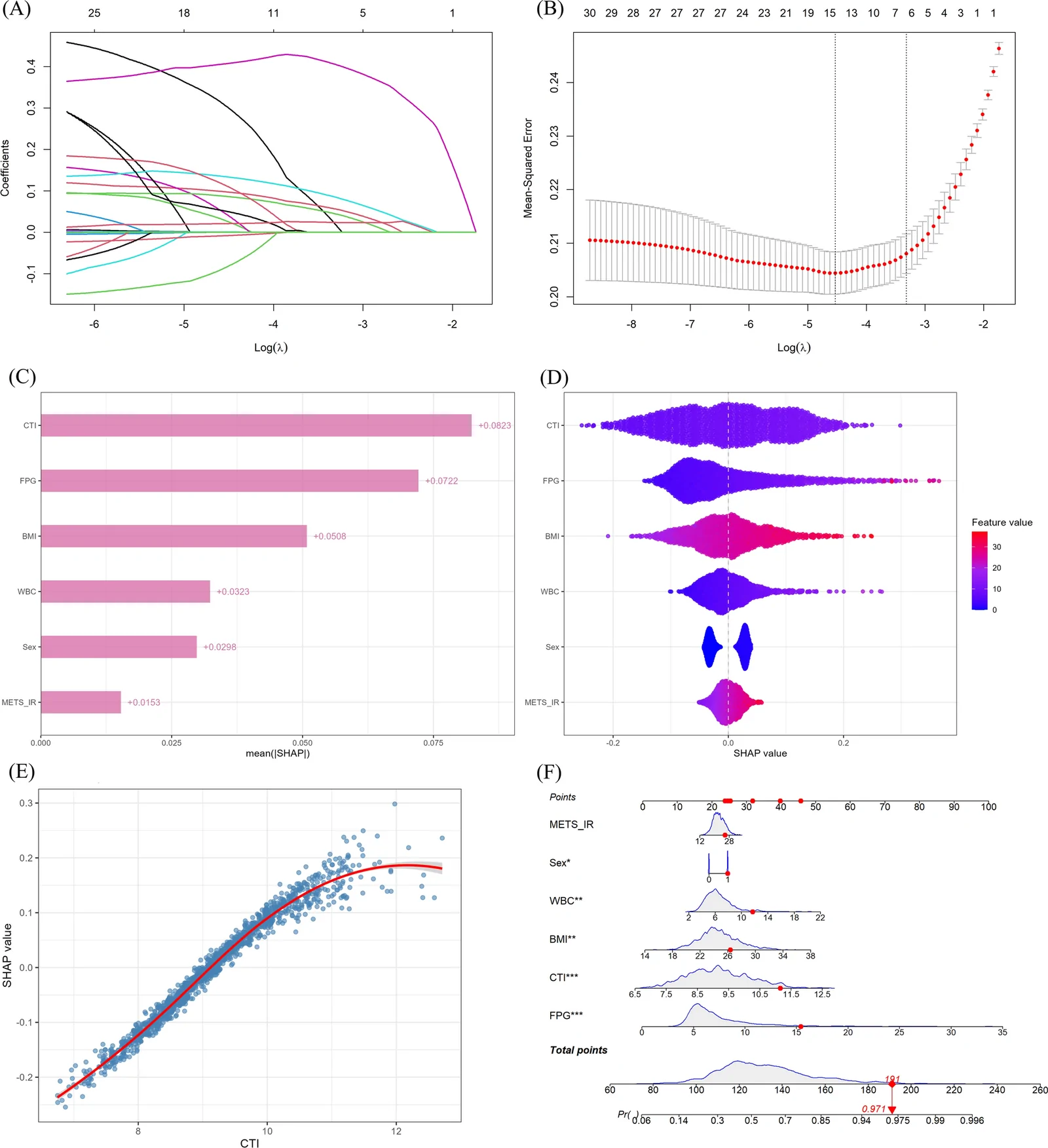
Screening of variables associated with coronary artery lesion severity. **A** Coefficient path plot of LASSO regression for variable screening; **B** Ten-fold cross-validation error curve to determine the optimal penalty λ value; **C** SHAP feature importance bar chart of the LASSO-selected logistic regression model; **D** SHAP beeswarm plot revealing the influence direction and intensity of each variable; **E** SHAP dependence curve demonstrating the nonlinear association between CTI and coronary lesion risk; **F** Nomogram model for individualized risk assessment of coronary stenosis severity. CTI, C-reactive protein-triglyceride-glucose index; FPG, fasting plasma glucose; BMI, body mass index; WBC, white blood cell; METS_IR, metabolic insulin resistance index

This study employed feature selection methods to systematically evaluate the strength of associations between multiple variables and the degree of coronary artery stenosis. First, LASSO regression was used to screen for key variables associated with the degree of coronary artery stenosis. Coefficient path plot (Fig. 2A) and tenfold cross‑validation curve (Fig. 2B) illustrate the entire variable selection process. Based on the optimal λ penalty value determined by cross‑validation, six independent associated variables with non‑zero coefficients were ultimately selected, including CTI, FPG, BMI, sex, WBC, and the METS‑IR index. The VIF values indicated no collinearity among these six variables (Table S2). To further elucidate the patterns of association between each factor and the severity of coronary artery stenosis, SHAP analysis was performed in this study. Figure 2C quantifies and ranks the contribution of each variable based on the mean absolute SHAP value, confirming that CTI has the strongest association with the outcome in the model. Figure 2D presents the SHAP beeswarm plot, where the color gradient from blue to red represents increasing feature values from low to high, and the vertical axis displays variables in descending order of mean absolute SHAP values (indicating importance ranking). The plot illustrates the association between each indicator’s value and its SHAP value: higher levels of CTI, FPG, BMI, WBC, and METS‑IR positively increase the risk of coronary artery stenosis, with CTI and FPG showing a non‑linear saturation effect. In addition, male sex contributes positively to the SHAP value, indicating that males have a higher baseline risk of coronary stenosis than females. The SHAP dependence plots (Fig. 2E and Figure S1) further refine the association patterns between each independent variable and coronary stenosis: CTI and FPG exhibit a rapid increase in association at lower levels, followed by a plateau at higher levels, demonstrating a non‑linear characteristic; in contrast, METS‑IR, WBC, and BMI show a stable linear positive association with coronary stenosis. Based on the above six most strongly associated factors, this study constructed an individualized risk nomogram (Fig. 2F) to visually quantify the relative contribution of each indicator to the degree of coronary artery stenosis, thereby enabling visualized risk stratification for high‑risk populations.

### Associations between CTI and CAD severity in patients with different types of hypertension

#### Associations between CTI and CAD severity in patients with hypertension

**Fig. 3:**
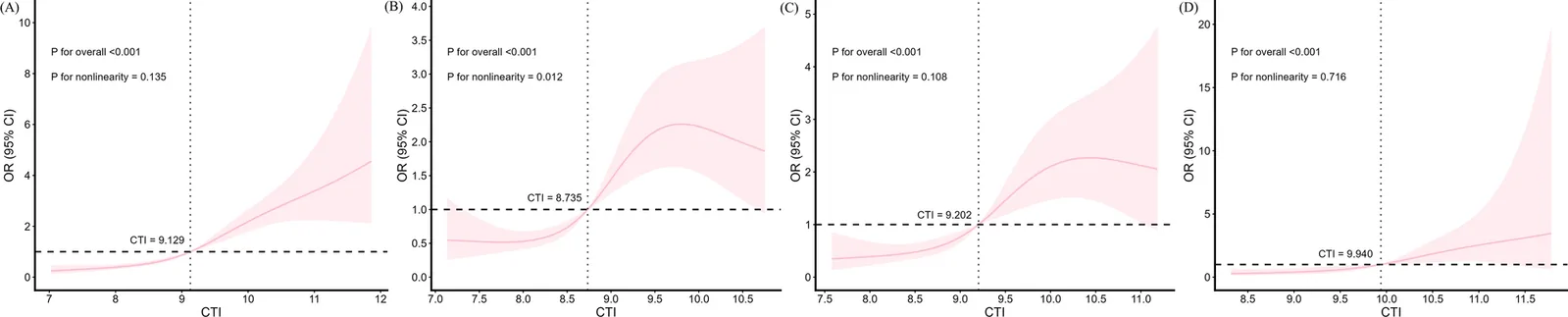
Restricted cubic spline curves for the association between CTI and multi-vessel CAD after covariate adjustment in hypertensive patients, overall and by glucose metabolic states. **A** Over all; **B** NGR group patients, **C** pre-DM group patients, **D** DM group patients. OR, odds ratios; CTI C-reactive protein-triglyceride-glucose index; NGR, normal glucose regulation; Pre-DM, pre-diabetes mellitus; DM, diabetes mellitus; CAD, coronary artery disease

As shown in Table 3 and Table S3, logistic regression models revealed that CTI was significantly associated with CAD severity both before and after multivariate adjustment (*P* < 0.001). When CTI was analyzed as a continuous variable, it was significantly associated with multi‑vessel CAD (OR = 2.155, 95% CI 1.839–2.455, *P* < 0.001) and also significantly associated with moderate‑to‑severe stenosis (OR = 1.818, 95% CI 1.603–2.062, *P* < 0.001). When CTI was used as a categorical variable, after adjustment for confounders, patients in the T3 group had a 5.747‑fold higher risk of multi‑vessel CAD compared with those in the T1 group (95% CI 4.283–7.712, *P* < 0.001), and the risk of moderate‑to‑severe coronary stenosis in the T3 group was 3.763 times that of the T1 group (95% CI 2.819–5.025, *P* < 0.001). Multiple linear regression analysis showed that CTI was significantly positively associated with ln(Gensini score + 1) across all three models (all *P* < 0.001). In the fully adjusted model, each 1-unit increase in CTI was associated with a 0.263-unit increase in the log-transformed Gensini score (B = 0.263, 95% CI: 0.221–0.306) (Table S4). Restricted cubic spline (RCS) analysis showed (Fig. 3A, Figure S2A) that, after adjustment for confounders, there was a linear dose‑response relationship between CTI and the risk of multi‑vessel CAD as well as moderate‑to‑severe coronary stenosis (overall *P* < 0.001, *P* for non‑linearity > 0.05).

**Table 3:** Association between CTI and multi-vessel CAD in patients with hypertension

Variables	Model 1		Model 2		Model 3	
	OR (95%CI)	*P* value	OR (95%CI)	*P* value	OR (95%CI)	*P* value
CTI	2.245 (1.977–2.550)	< 0.001	2.155 (1.839–2.454)	< 0.001	2.155 (1.839–2.455)	< 0.001
CTI T1	Reference		Reference		Reference	
CTI T2	2.724 (2.086–3.556)	< 0.001	2.662 (2.021–3.505)	< 0.001	2.668 (2.024–3.516)	< 0.001
CTI T3	6.133 (4.605–8.167)	< 0.001	5.736 (4.277–7.692)	< 0.001	5.747 (4.283–7.712)	< 0.001

Model 1: unadjusted

Model 2: adjusted for sex, age, BMI, SBP, DBP, smoking and drinking

Model 3: adjusted for the same variables as model 2, with additional covariates, including CCB, statin, antiplatelet, beta blockers and ACEI/ARB

CTI, C-reactive protein-triglyceride-glucose index; CAD, coronary artery disease; OR, odds ratio; CI confidence interval; BMI, body mass index; SBP, systolic blood pressure; DBP, diastolic blood pressure

**Fig. 4:**
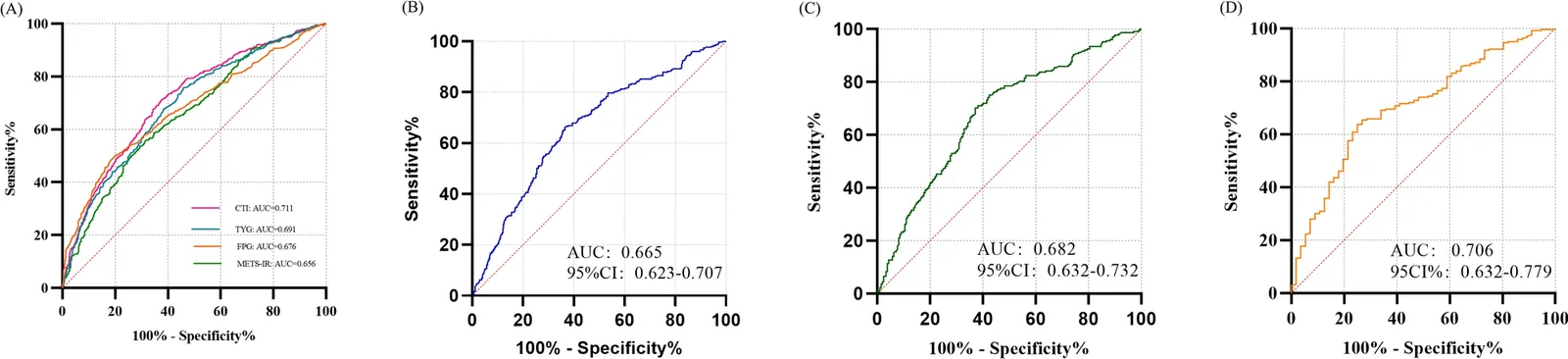
ROC curve analysis of CTI for detecting multi-vessel CAD in hypertension patients: overall and by glucose metabolism status. **A** Overall; **B** NGR group patients; **C** Pre-DM group patients; **D** DM group patients. AUC, Area under the curve; CI, confidence interval

ROC curve analysis was performed to evaluate the discriminative value of CTI for the severity of CAD. The results showed that the area under the curve (AUC) of CTI for discriminating multi‑vessel CAD was 0.711 (95% CI 0.683–0.738, *P* < 0.001), with an optimal cut‑off value of 8.977, a corresponding sensitivity of 0.713, a specificity of 0.625, and a Youden index of 0.338 (Fig. 4A, Table 7). The AUC of CTI for discriminating moderate‑to‑severe coronary stenosis was 0.673 (95% CI 0.644–0.702, *P* < 0.001) (Table S8).

#### Associations between CTI and CAD severity in patients with H-type hypertension

Regarding the joint effect of CTI and Hcy levels on the risk of coronary artery disease severity (Figure S3), compared with Hcy < 15 μmol/L and CTI T1, patients with Hcy ≥ 15 μmol/L and CTI T3 had the highest risk of multi-vessel coronary artery disease (OR: 4.570, 95% CI: 3.093–6.754). Multivariate logistic regression analysis showed that, there was a significant multiplicative interaction between CTI and hypertension subtypes on the risk of multi-vessel CAD (P for interaction = 0.033) (Table 4). Among patients with H‑type hypertension, CTI was significantly and positively correlated with the severity of CVD. When CTI was analyzed as a continuous variable, after adjustment for confounders, each one‑unit increase in CTI was associated with a 121.3% higher risk of multi‑vessel disease (OR = 2.213, 95% CI 1.862–2.629, *P* < 0.001) and an 80.6% higher risk of moderate‑to‑severe coronary stenosis (OR = 1.806, 95% CI 1.531–2.132, *P* < 0.001). Using T1 as the reference, both the T2 and T3 groups showed significantly increased risks of multi‑vessel disease and moderate‑to‑severe coronary stenosis, with the strongest associations observed in the T3 group: the OR for multi‑vessel disease was 6.092 (95% CI 4.144–8.955, *P* < 0.001), and the OR for moderate‑to‑severe coronary stenosis was 3.525 (95% CI 2.425–5.123, *P* < 0.001) (Table S5).

**Table 4:** Association between CTI and Multi-vessel CAD in different type hypertension patients

Characterics	Variables	Model 1		Model 2			Model 3		*P* for interaction
		OR (95%CI)	*P* value	OR (95%CI)		*P* value	OR (95%CI)	*P* value	
Non-H-type hypertension	CTI	1.436 (1.210–1.703)	< 0.001	1.353 (1.133–1.616)	0.001		1.342 (1.123–1.604)	0.001	0.033
	CTI T1	Reference		Reference			Reference		
	CTI T2	1.421 (0.954–2.116)	0.084	1.356 (0.893–2.060)	0.153		1.341 (0.880–2.043)	0.172	
	CTI T3	1.947 (1.276–2.973)	0.002	1.686 (1.086–2.620)	0.020		1.655 (1.063–2.576)	0.023	
H-type hypertension	CTI	2.224 (1.882–2.628)	< 0.001	2.225 (1.873–2.643)	< 0.001		2.213 (1.862–2.629)	< 0.001	
	CTI T1	Reference		Reference			Reference		
	CTI T2	3.282 (2.282–4.720)	< 0.001	3.402 (2.335–4.958)	< 0.001		3.376 (2.311–4.932)	< 0.001	
	CTI T3	5.949 (4.109–8.614)	< 0.001	6.127 (4.179–8.985)	< 0.001		6.092 (4.144–8.955)	< 0.001	

Model 1: unadjusted

Model 2: adjusted for sex, age, BMI, SBP, DBP, smoking and drinking

Model 3: adjusted for the same variables as model 2, with additional covariates, including CCB, statin, antiplatelet, beta blockers and ACEI/ARB

CTI, C-reactive protein-triglyceride-glucose index; CAD, coronary artery disease; OR, odds ratio; CI, confidence interval; BMI, body mass index; SBP, systolic blood pressure; DBP, diastolic blood pressure

**Fig. 5:**
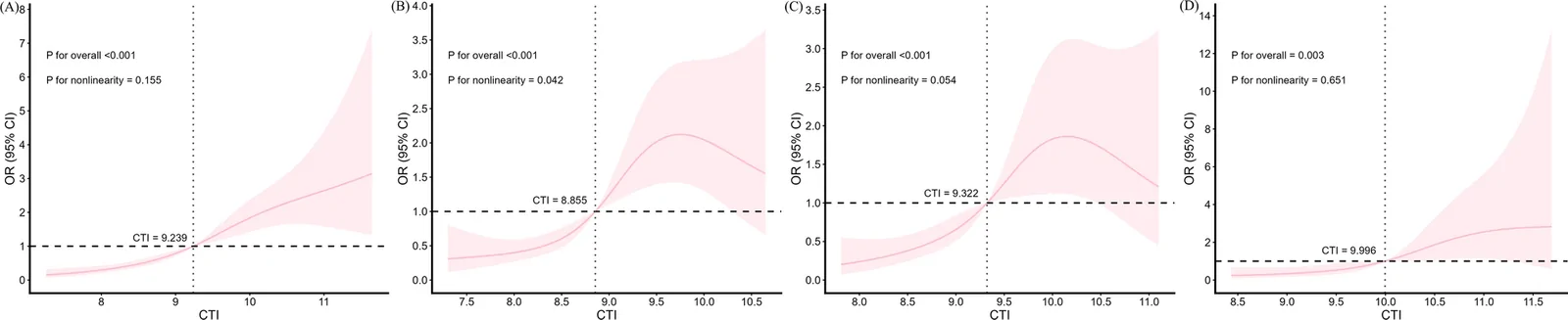
Restricted cubic spline curves for the association between CTI and multi-vessel CAD after covariate adjustment in H-type hypertension, overall and by glucose metabolic states. **A** Over all; **B** NGR group patients; **C** pre-DM group patients; **D** DM group patients. OR, odds ratios; CTI C-reactive protein-triglyceride-glucose index; NGR, Normal glucose regulation; pre-DM, pre-diabetes mellitus; DM, diabetes mellitus; CAD, coronary artery disease

RCS analysis showed (Fig. 5A, Figure S4A) that, after adjustment for confounders, there was a linear dose‑response relationship between CTI and both multi‑vessel disease (overall *P* < 0.001, *P* for non‑linearity = 0.155) and moderate‑to‑severe coronary stenosis (overall *P* < 0.001, *P* for non‑linearity = 0.660).

**Fig. 6:**
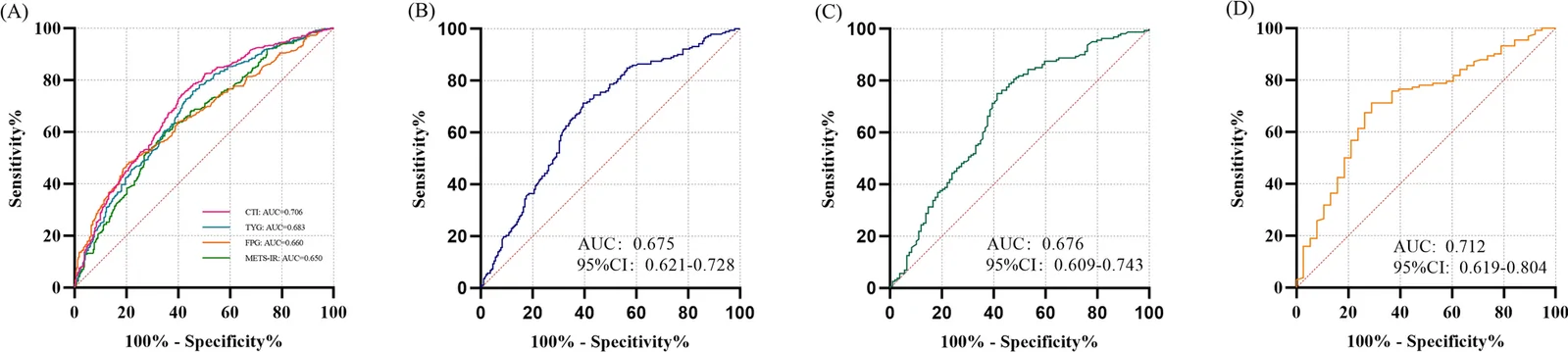
ROC curve analysis of CTI for detecting multi-vessel CAD in H-type hypertension patients: overall and by glucose metabolism status. **A** Overall; **B** NGR group patients; **C** pre-DM group patients; **D** DM group patients. AUC, Area under the curve; CI, confidence interval

ROC curve analysis (Fig. 6A, Table S8) indicated that, in the H‑type hypertension population, the AUC of CTI for evaluating its association with multi‑vessel disease was 0.706 (95% CI 0.670–0.742, *P* < 0.001), and the AUC for evaluating its association with moderate‑to‑severe stenosis was 0.661 (95% CI 0.621–0.700, *P* < 0.001).

### Associations between CTI and CAD severity in different glucose metabolism states

#### Associations between CTI and CAD severity in different glucose metabolism states in patients with hypertension

In this study of hypertensive patients, binary logistic regression analysis to evaluate the influence of glucose metabolic statuses on the relationship between CTI and CAD severity (Table 5, Table S6). When CTI was analyzed as a continuous variable in the three subgroups, it showed a significant positive correlation with CAD severity in all subgroups. Specifically, among hypertensive patients with DM, each one‑unit increase in CTI was associated with a 1.260‑fold increase in the risk of multi‑vessel CAD (OR = 2.260, 95% CI 1.578–3.237, *P* < 0.001) and a 0.721‑fold increase in the risk of moderate‑to‑severe coronary stenosis (OR = 1.721, 95% CI 1.208–2.451, *P* = 0.003). When T1 was used as the reference, no statistically significant association between CTI and multi‑vessel CAD was found in T2 (*P* = 0.381). However, in the T3 group, hypertensive patients with comorbid diabetes had a 5.254‑fold higher risk of multi‑vessel CAD (OR = 5.254, 95% CI 2.092–13.195, *P* < 0.001). Notably, among hypertensive patients with NGR and Pre-DM, there was a statistically significant association between CTI and moderate‑to‑severe coronary stenosis (*P* < 0.001). However, among hypertensive patients with DM, no significant association was found between CTI and moderate‑to‑severe coronary stenosis (OR = 2.582, 95% CI 0.949–7.029, *P* > 0.05).

**Table 5:** Associations between CTI and Multi-vessel CAD according to different glucose metabolism states in patients with hypertension

Glucose metabolism state	Variables	Model 1		Model 2		Model 3	
		OR (95%CI)	*P* value	OR (95%CI)	*P* value	OR (95%CI)	*P* value
NGR	CTI	1.931 (1.591–2.343)	< 0.001	1.815 (1.485–2.217)	< 0.001	1.804 (1.476–2.206)	< 0.001
	CTI T1	Reference		Reference		Reference	
	CTI T2	2.930 (2.051–4.185)	< 0.001	2.828 (1.949–4.102)	< 0.001	2.827 (1.944–4.112)	< 0.001
	CTI T3	3.852 (2.469–6.010)	< 0.001	3.571 (2.247–5.674)	< 0.001	3.534 (2.221–5.624)	< 0.001
PRE-DM	CTI	2.028 (1.612–2.550)	< 0.001	1.972 (1.557–2.498)	< 0.001	1.984 (1.560–2.525)	< 0.001
	CTI T1	Reference		Reference		Reference	
	CTI T2	2.376 (1.455–3.882)	0.001	2.441 (1.456–4.094)	0.001	2.524 (1.486–4.287)	0.001
	CTI T3	4.536 (2.714–7.580)	< 0.001	4.511 (2.644–7.696)	< 0.001	4.642 (2.688–8.015)	< 0.001
DM	CTI	2.252 (1.590–3.189)	< 0.001	2.229 (1.564–3.177)	< 0.001	2.260 (1.578–3.237)	< 0.001
	CTI T1	Reference		Reference		Reference	
	CTI T2	1.519 (0.617–3.744)	0.363	1.491 (0.594–3.738)	0.395	1.524 (0.594–3.911)	0.381
	CTI T3	5. 059 (2.101–12.818)	< 0.001	4.976 (2.023–12.236)	< 0.001	5.254 (2.092–13.195)	< 0.001

Model 1: unadjusted

Model 2: adjusted for Sex, age, BMI, SBP, DBP, Smoking and Drinking

Model 3: adjusted for the same variables as model 2, with additional covariates, including CCB, statin, antiplatelet, beta blockers and ACEI/ARB CTI C-reactive protein-triglyceride-glucose index; CAD Coronary Artery Disease; OR odds ratio; CI confidence interval; BMI body mass index; SBP systolic blood pressure; DBP diastolic blood pressure

RCS analysis revealed that the dose‑response relationship between CTI and CAD severity differed across glucose metabolic statuses in this hypertensive cohort (Fig. 3B–D, Figure S2B–D). In the Pre‑DM group (overall *P* < 0.001, *P* for non‑linearity = 0.108) and the DM group (overall *P* < 0.001, *P* for non‑linearity = 0.716), a linear dose‑response relationship was observed between CTI and the risk of multi‑vessel CAD. In contrast, a non‑linear dose‑response relationship was found in the NGR group (overall *P* < 0.001, *P* for non‑linearity = 0.012), with a cut‑off value of 9.940. Under different glucose metabolism statuses, the dose‑response relationship between CTI and the risk of moderate‑to‑severe coronary stenosis was consistently linear (overall *P* < 0.05, *P* for non‑linearity > 0.05).

CTI effectively discriminated CAD severity across all glucose metabolism statuses in this hypertensive population (Fig. 4B–D, Table 7, Table S8). Specifically, in the DM group, the AUC of CTI for discriminating multi‑vessel CAD was 0.706 (95% CI 0.632–0.779, *P* < 0.001), with an optimal cut‑off value of 9.724, a sensitivity of 0.638, a specificity of 0.750, and a Youden index of 0.388. The AUC for discriminating moderate‑to‑severe coronary stenosis was 0.650 (95% CI 0.567–0.733, *P* < 0.001). These findings suggest that CTI has better discriminative ability in diabetic patients within this hypertensive cohort.

#### Associations between CTI and CAD severity in different glucose metabolism states in patients with H-type hypertension

As shown in Table 6 and Table S7, CTI as a continuous variable was significantly positively correlated with CVD severity in all three subgroups of different glucose metabolic statuses (*P* < 0.001). Among these, the DM subgroup had the highest OR values: the OR for multi‑vessel disease was 2.433 (95% CI 1.503–3.938, *P* < 0.001), and the OR for moderate‑to‑severe coronary stenosis was 1.933 (95% CI 1.109–3.138, *P* < 0.001). Using T1 as the reference, T3 significantly increased the risk of multi‑vessel disease in all subgroups, with the greatest risk increase observed in the DM subgroup (OR = 6.475, 95% CI 1.693–24.773, *P* = 0.006). Notably, in the DM group, CTI T2 was not significantly associated with the risk of multi‑vessel disease (OR = 1.577, 95% CI 0.389–6.400, *P* = 0.527). In addition, no statistically significant association was found between CTI and the risk of moderate‑to‑severe coronary stenosis within the DM group, whereas this association was significant in the NGR and Pre‑DM groups (*P* < 0.001). This finding is consistent with the results of the DM subgroup analysis in the hypertensive population.

**Table 6:** Associations between CTI and multi-vessel CAD according to different glucose metabolism states in patients with H-type hypertension

Glucose metabolism state	Variables	Model 1		Model 2		Model 3	
		OR (95%CI)	*P* value	OR (95%CI)	P value	OR (95%CI)	*P* value
NGR	CTI	2.063 (1.596–2.667)	< 0.001	2.037 (1.555–2.668)	< 0.001	2.023 (1.542–2.654)	< 0.001
	CTI T1	Reference		Reference		Reference	
	CTI T2	3.638 (2.249–5.886)	< 0.001	3.769 (2.266–6.269)	< 0.001	3.824 (2.284–6.400)	< 0.001
	CTI T3	3.727 (2.169–6.405)	< 0.001	3.871 (2.184–6.862)	< 0.001	3.862 (2.168–6.878)	< 0.001
PRE-DM	CTI	1.981 (1.466–2.677)	< 0.001	2.057 (1.503–2.815)	< 0.001	2.016 (1.462–2.779)	< 0.001
	CTI T1	Reference		Reference		Reference	
	CTI T2	3.284 (1.671–6.456)	0.001	3.658 (1.794–7.461)	< 0.001	3.577 (1.711–7.482)	0.001
	CTI T3	5.268 (2.638–10.523)	< 0.001	6.051 (2.935–12.476)	< 0.001	5.843 (2.757–12.387)	< 0.001
DM	CTI	2.243 (1.461–3.444)	< 0.001	2.271 (1.439–3.586)	< 0.001	2.433 (1.503–3.938)	< 0.001
	CTI T1	Reference		Reference		Reference	
	CTI T2	1.314 (0.369–4.679)	0.673	1.304 (0.338–5.034)	0.700	1.577 (0.389–6.458)	0.527
	CTI T3	5.143 (1.541–7.165)	0.008	5.179 (1.423–8.847)	0.013	6.475 (1.693–24.773)	0.006

Model 1: unadjusted

Model 2: adjusted for sex, age, BMI, SBP, DBP, smoking and drinking

Model 3: adjusted for the same variables as model 2, with additional covariates, including CCB, statin, antiplatelet, beta blockers and ACEI/ARB

CTI, C-reactive protein-triglyceride-glucose index; CAD, coronary artery disease; OR, odds ratio; CI, confidence interval; BMI, body mass index; SBP, systolic blood pressure; DBP, diastolic blood pressure

RCS analysis (Figs. 5B–D and Figures S4B–D) revealed the dose‑response relationship between CTI and CVD severity under different glucose metabolism statuses in patients with H‑type hypertension. The dose‑response relationship between CTI and multi‑vessel disease varied across different glucose metabolism statuses: a non‑linear dose‑response relationship was observed in the NGR group (overall *P* < 0.001, *P* for non‑linearity = 0.042) with a cut‑off value of 8.855, whereas linear dose‑response relationships were observed in the Pre‑DM group (overall *P* < 0.001, *P* for non‑linearity = 0.054) and the DM group (overall *P* = 0.003, *P* for non‑linearity = 0.05). In contrast, the relationship between CTI and moderate‑to‑severe coronary stenosis was linear across all glucose metabolism subgroups (overall *P* < 0.005, *P* for non‑linearity > 0.05).

The ROC analysis shown in Figs. 6B–D illustrates the ability of CTI to discriminate multi‑vessel CAD in patients with H‑type hypertension under different glucose metabolic statuses. The AUC values for the NGR and Pre‑DM groups were 0.675 and 0.676, respectively. The AUC of CTI for discriminating multi‑vessel disease in the DM group was 0.712 (95% CI 0.619–0.804, *P* < 0.001), with an optimal cut‑off value of 9.674 (Table 7); The AUC of CTI for discriminating moderate‑to‑severe coronary stenosis in the DM group was 0.666 (95% CI 0.562–0.770, *P* = 0.004) (Table S8).

**Table 7:** The diagnostic value of CTI in assessing the risk of coronary artery stenosis in different populations

Test variables	AUC	SE	95%CI	*P* value	Best truncation value	Youden’s index	sensitivity	specificity
ALL	0.711	0.014	0.683–0.738	< 0.001	8.977	0.338	0.713	0.625
NGR	0.655	0.021	0.623–0.707	< 0.001	8.714	0.297	0.666	0.631
Pre-DM	0.682	0.026	0.632–0.732	< 0.001	9.137	0.331	0.702	0.629
DM	0.706	0.037	0.632–0.779	< 0.001	9.724	0.388	0.638	0.750
H-ALL	0.706	0.018	0.670–0.742	< 0.001	8.954	0.331	0.758	0.573
H-NGR	0.675	0.027	0.621–0.728	< 0.001	8.715	0.319	0.714	0.605
H-Pre-DM	0.676	0.034	0.609–0.743	< 0.001	9.137	0.337	0.750	0.587
H-DM	0.712	0.047	0.619–0.804	< 0.001	9.674	0.432	0.712	0.711

NGR, normal glucose regulation group in patients with hypertension; Pre-DM, pre-diabetes group in patients with hypertension; DM, diabetes group in patients with hypertension; H-NGR, normal glucose regulation group in patients with H-type hypertension; H-Pre-DM, pre-diabetes group in patients with H-type hypertension; H-DM, diabetes mellitus group in patients with H-type hypertension; ALL, all patients with hypertension; H-ALL, all patients with H-type hypertension

### Analytical assessment of the association between CTI and CAD severity

**Fig. 7:**
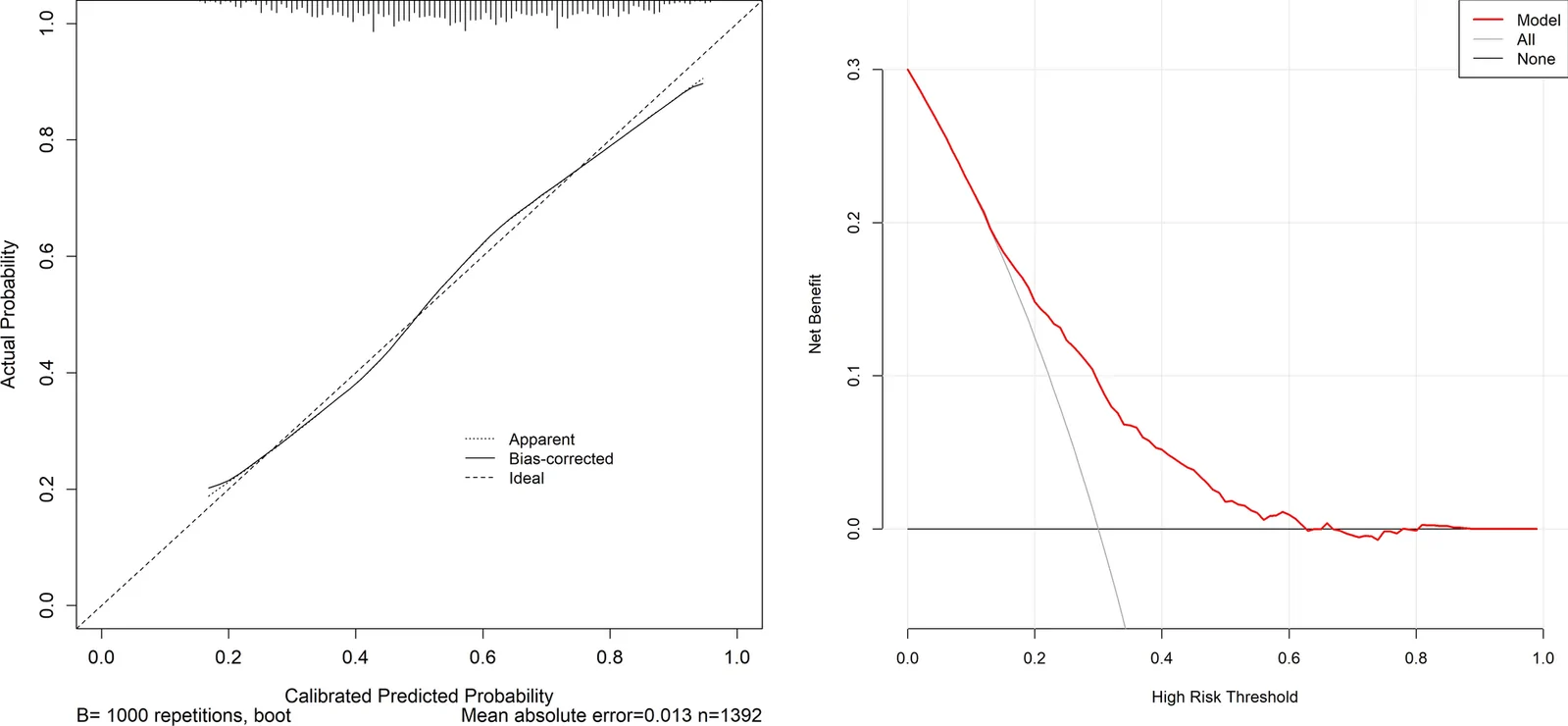
Assessment of CTI performance in distinguishing multi-vessel CAD. **A** calibration curve; **B** decision curve analysis (DCA)

Calibration curve and decision curve analysis (DCA) were used to evaluate the performance of CTI in assessing the severity of CAD. The calibration curve (Fig. 7A) showed that the fitted curve of the CTI‑based model was highly consistent with the ideal diagonal line, indicating good model calibration. DCA (Fig. 7B) demonstrated that, across a wide range of risk thresholds, the CTI‑based model provided a positive clinical net benefit and outperformed the “treat all” or “treat none” strategies, fully reflecting the clinical utility of CTI. In the overall hypertensive population and H-type subgroup, CTI showed higher AUC values than TyG, FPG, and METS-IR, indicating relatively better discriminative ability (Table S9). In the overall hypertensive population, adding CTI to the basic model (AUC = 0.706, 95% CI: 0.679–0.733) increased the AUC to 0.752 (95% CI: 0.726–0.777, *P* < 0.001), with an NRI of 0.548 (95% CI: 0.451–0.648, *P* < 0.001) and an IDI of 0.061 (95% CI: 0.049–0.075, *P* < 0.001). In the H-type hypertension population, the basic model had a lower AUC of 0.679 (95% CI: 0.642–0.716), but CTI raised it to 0.742 (95% CI: 0.708–0.777) with more substantial improvements (NRI = 0.585, 95% CI: 0.453–0.726, *P* < 0.001, IDI = 0.081, 95% CI: 0.062–0.100, *P* < 0.001). Compared with TyG, METS‑IR, and FPG, CTI exhibited the best performance in both populations, with a particularly pronounced relative advantage in the H‑type hypertension population (Table S10).

### Subgroup analysis

**Fig. 8:**
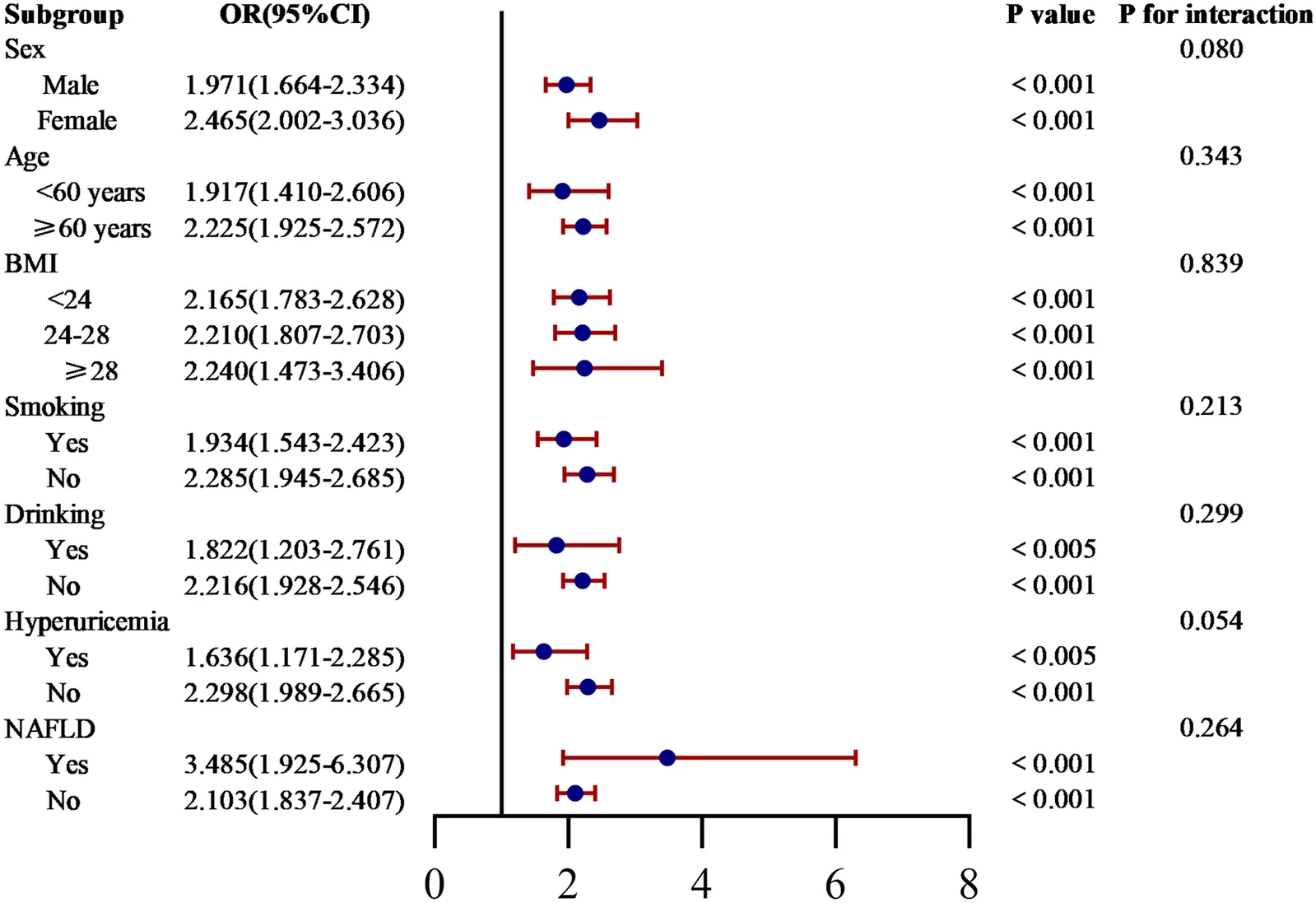
Subgroup analyses of the effect of the CTI on the incidence of multi-vessel CAD in hypertension patients. Each subgroup analysis was adjusted for sex, age, BMI, SBP, DBP, smoking, drinking, CCB, statin, antiplatelet, beta blockers and ACEI/ARB

To evaluate whether the association between CTI and CAD severity varied according to key demographic and clinical characteristics, we performed subgroup analyses (Fig. 8, Figure S5). The results showed that higher CTI levels were positively associated with increased CAD severity across all subgroups. After stratification by age, sex, BMI, smoking history, drinking history, hyperuricemia, and non‑alcoholic fatty liver disease, none of the interaction tests for the association between CTI and CAD severity reached statistical significance (all *P* for interaction > 0.05), indicating that the association between CTI and CAD severity was generally consistent across these population subgroups without significant heterogeneity.

## Discussion

This study demonstrated that CTI is significantly associated with the severity of CAD in patients with hypertension and the RCS analysis revealed a linear correlation between CTI and the severity of CAD. This association was substantially stronger and statistically significant in individuals with H-type hypertension, with a significant interaction between CTI and H-type hypertension on CAD severity.

Hypertension and diabetes are important and common risk factors for cardiovascular disease, and they can act synergistically to accelerate the development and progression of coronary heart disease. H‑type hypertension is a specific type of hypertension that, compared with essential hypertension, significantly increases the risk of coronary artery disease and adversely affects the prognosis of patients with coronary artery disease [2, 44]. Controlling blood pressure and blood glucose is of paramount importance in the secondary prevention of cardiovascular disease. Therefore, early identification of such patients and assessment of their risk of coronary artery stenosis are particularly important. The association between CTI and coronary artery stenosis has a clear pathophysiological basis. Chronic low‑grade inflammation promotes atherosclerotic plaque instability, endothelial injury, and thrombosis through activation of the NF‑κB signaling pathway [45, 46]. CRP is not only a sensitive marker of systemic inflammation but also directly exacerbates insulin resistance by inhibiting IRS-1 phosphorylation [29]. At the same time, hypertriglyceridemia exacerbates lipotoxicity, oxidative stress, and ectopic lipid deposition, further impairs insulin signaling and vascular homeostasis, and also increases the risk of thrombosis by affecting platelet function, the coagulation system, and endothelial function [47]. Persistent hyperglycemia exacerbates myocardial ischemia‑reperfusion injury by increasing oxidative stress, mitochondrial dysfunction, and the formation of advanced glycation end‑products [48]. The above mechanisms do not act in isolation but form a vicious cycle between IR‑driven lipotoxicity and inflammatory signal amplification. CTI integrates these three indicators into a single composite biomarker, reflecting the overall metabolic‑inflammatory burden [49].

A meta‑analysis confirmed that an elevated TyG index increases the risk of heart failure and coronary artery disease [50]. An elevated TyG index is associated with the severity of coronary artery disease in patients with coronary heart disease, and a high TyG index can identify CHD patients at high risk of multi‑vessel CAD [51]. The TyG index is correlated with the degree of coronary stenosis and can identify asymptomatic patients with type 2 diabetes mellitus (T2DM) who are at high risk of coronary stenosis [52]. Among patients with H‑type hypertension, the TyG index is significantly correlated with the degree of coronary artery lesions; after adjustment for relevant confounders, the TyG index also shows a significant correlation with the severity of coronary artery lesions [53]. Li et al. found that the mediating effect of inflammation on the association between the TyG index and multi‑vessel CAD was 31.66% [54]. Cui et al. demonstrated that the coexistence of high TyG index and high hsCRP levels was associated with an increased risk of cardiovascular disease (HR = 1.300, 95% CI 1.155–1.462), revealing the synergistic exposure effect and mutual mediating role of the TyG index and hsCRP in cardiovascular disease [29]. Xu et al. reported that CTI was significantly and linearly associated with the risk of coronary heart disease in the general population, and that each one‑unit increase in CTI was associated with a 35.7% higher risk of coronary heart disease, suggesting that CTI can serve as a linear indicator for assessing the prevalent risk of coronary heart disease [55]. Furthermore, CTI is significantly associated with the risk of recurrent cardiovascular events in patients undergoing percutaneous coronary intervention [56]. A multinational prospective study demonstrated that CTI was not only significantly associated with long‑term all‑cause mortality and cardiovascular mortality, but also that diabetes served as a major mediator of the association between elevated CTI and excess mortality risk [57]. The above findings indicate that CTI is a simple and effective biomarker of coronary artery disease and holds significant clinical value.

This study focused on the complex role of multiple comorbidities and their value for clinical translation, with particular attention to the risk of coronary artery stenosis in patients with comorbid H‑type hypertension. Our findings indicate that CTI is linearly and positively correlated with multi‑vessel disease, and patients with multi‑vessel disease have higher CTI values, which is consistent with the results of Gao et al. [56]. Moreover, this association was even more pronounced in patients with H‑type hypertension. This suggests that the interplay between metabolism and inflammation may exacerbate clinical deterioration in the context of comorbidity. Our analysis further revealed a significant joint effect of CTI and homocysteine on the risk of multi-vessel CAD. Previous studies have shown that elevated homocysteine is associated with the severity of coronary stenosis, Gensini score, and multi-vessel disease, suggesting that it may reflect endothelial dysfunction, oxidative stress, and atherosclerotic burden [7, 58]. The combined assessment of CTI and Hcy may provide a more comprehensive coverage of key pathological pathways, including inflammation, metabolic disturbance, and endothelial injury, compared with either indicator alone, thereby improving the identification of coronary artery disease risk in patients with H-type hypertension [35, 49, 59–61]. Although H-type hypertension is commonly defined as hypertension accompanied by Hcy levels > 10 μmol/L according to previous consensus recommendations, the optimal Hcy threshold for cardiovascular risk stratification remains uncertain. Therefore, the findings of the present study should be interpreted within the context of the operational definition adopted, and further prospective studies comparing different Hcy thresholds are needed to determine the most clinically appropriate definition.

The association between CTI and multi-vessel CAD varied across different glucose metabolic statuses, with a more pronounced association observed in patients with diabetes mellitus (DM). Among patients with H-type hypertension and coexisting DM, this association reached statistical significance. In the DM subgroup, the association between CTI and moderate-to-severe coronary stenosis showed a trend consistent with that in the overall population (OR > 1), but did not reach statistical significance (*P* > 0.05). Sensitivity power analysis for this subgroup indicated that the power for the moderate-to-severe stenosis analysis was relatively insufficient. Therefore, this null finding may be largely limited by sample size, and the above non-significant results should be interpreted with caution. Similar phenomena have been reported in previous studies. Huo et al. investigated the association between the TyG‑CRP index and stroke risk under different glucose metabolism statuses and found that the strong association existed only in individuals with normal glucose regulation or pre‑diabetes, but was not observed in patients with diabetes [62]. Using the NHANES cohort, Sun et al. confirmed that CTI was linearly and positively associated with cardiovascular mortality, overall CVD prevalence, and all‑cause mortality. However, a significant association between elevated CTI levels and increased CVD risk was observed only in the non‑diabetic population, whereas this association was not detected in the diabetic population [63]. The nonlinear association observed in the NGR subgroup in the restricted cubic spline analysis should be interpreted with caution. The curve shape may be partially attributable to differences in sample size and statistical power across subgroups, rather than necessarily reflecting true biological heterogeneity. Based on the current data, we are unable to distinguish between these possibilities. Therefore, our findings should be regarded as exploratory and require further validation in larger, prospective studies.

Our findings suggest that CTI may serve as a useful composite indicator for assessing the severity of coronary artery disease in H-type hypertensive patients. In the ROC analysis, CTI showed a higher AUC for evaluating the association with the degree of coronary stenosis compared with FPG, TyG, and METS-IR, with the differences reaching statistical significance. When CTI was added to a basic model comprising conventional risk factors, both the AUC and reclassification indices (NRI and IDI) improved in the H-type hypertensive population, suggesting that CTI offers unique incremental value for risk assessment in H‑type hypertension population. This is consistent with previous research findings on other composite indicators. In predicting incident coronary heart disease, CTI demonstrated stronger predictive ability than traditional metabolic indicators (TG, FPG) and the TyG index alone [64]. Studies have confirmed that CTI significantly outperforms CRP or the TyG index alone in predicting CVD outcomes [63]. These findings indicate that CTI, which integrates inflammatory and metabolic markers, provides more comprehensive risk prediction and stratification information for coronary artery stenosis than any single indicator alone. The traditional TyG index can reflect insulin resistance (IR) but does not capture inflammatory mechanisms, making it difficult to capture the synergistic effect between IR and chronic inflammation. By integrating CRP and TyG, CTI simultaneously reflects the dual pathophysiological processes of inflammation and IR, thereby providing a new perspective for risk stratification of coronary stenosis. CTI is calculated based on routine laboratory parameters, making it practical and easy to promote. It should be regarded as a complementary comprehensive biomarker to TyG rather than a replacement. Its core value lies in capturing the inflammatory information that TyG cannot reflect, thereby reflecting the overall metabolic‑inflammatory burden. In the subgroups of multi‑vessel disease and moderate‑to‑severe stenosis, CTI levels show a significant linear positive correlation with the degree of coronary stenosis, which helps in the early identification of high‑risk individuals.

### Limitations

First, this study is a retrospective, single-center, cross-sectional observational study, thereby precluding the establishment of a definitive causal relationship between the CTI and severe stenosis. All participants were hospitalized patients who underwent coronary angiography based on clinical indications, which introduces inherent referral bias and limits the generalizability of the findings. Moreover, the optimal cut-off value of CTI proposed in this study is data-dependent and represents an exploratory result; its general applicability requires further validation in multicenter, prospective, large-sample populations. Second, there is currently no universally accepted clinical cut-off value for the Gensini score. The stratification of moderate-to-severe stenosis in this study was based on tertiles within the cohort, and this cut-off was derived from the statistical distribution characteristics. Therefore, the clinical interpretability and cross-population generalizability of this classification approach are limited. Third, due to the inherent limitations of retrospective data collection, this study did not obtain complete information on confounding factors such as specific glucose-lowering regimens, precise duration of hypertension, and long-term blood pressure control status. Although we adjusted for multiple potential confounders, the possibility of residual confounding cannot be fully excluded.

## Conclusion

This retrospective study confirmed that elevated CTI levels were significantly associated with the severity of CAD in patients with hypertension, and this association remained independent after adjustment for traditional risk factors. More importantly, this association was particularly prominent in patients with H‑type hypertension. In conclusion, CTI, as a composite inflammatory and metabolic biomarker, offers complementary value in cardiovascular risk stratification, particularly for early intervention in patients with H-type hypertension; however, it should be regarded as an adjunctive tool rather than a universally superior biomarker. However, these findings are exploratory and require validation in larger, prospective cohorts. At the same time, the impact of dynamic changes in CTI over time on the progression of coronary stenosis should be explored, and whether CTI‑guided intervention strategies can improve patient outcomes needs to be evaluated.

## Supplementary Information

Additional file 1

## Data Availability

No datasets were generated or analysed during the current study.

## Abbreviations

ADA: American diabetes association
AGEs: Advanced glycation end-products
ALT: Alanine aminotransferase
AST: Aspartate aminotransferase
AUC: Area under the curve
BMI: Body mass index
CAD: Coronary artery disease
CHD: Coronary heart disease
CI: Confidence interval
CKM: Cardiovascular-kidney-metabolic
CRP: C-reactive protein
CTI: C-reactive protein–triglyceride glucose index
CVD: Cardiovascular disease
DCA: Decision curve analysis
DM: Diabetes mellitus
DBP: Diastolic blood pressure
FPG: Fasting plasma glucose
HbA1c: Glycated hemoglobin A1c
HDL-C: High-density lipoprotein cholesterol
Hcy: Homocysteine
IDI: Integrated discrimination improvement
IL-6: Interleukin-6
IR: Insulin resistance
IQR: Interquartile range
IRS-1: Insulin receptor substrate-1
LASSO: Least absolute shrinkage and selection operator
LDL-C: Low-density lipoprotein cholesterol
METS-IR: Metabolic score for insulin resistance
NAFLD: Non-alcoholic fatty liver disease
NF-κB: Nuclear factor-kappa B
NHANES: National health and nutrition examination survey
NGR: Normal glucose regulation
NEUT: Neutrophil
NRI: Net reclassification improvement
OR: Odds ratio
PCI: Percutaneous coronary intervention
PLT: Platelet
Pre-DM: Pre-diabetes mellitus
RBC: Red blood cell
RCS: Restricted cubic spline
ROC: Receiver operating characteristic
SBP: Systolic blood pressure
SD: Standard deviation
SHAP: Shapley additive explanations
TC: Total cholesterol
TG: Triglycerides
TyG: Triglyceride-glucose index
UA: Uric acid
VIF: Variance inflation factor
WBC: White blood cell
