# Supplementary material for: C-reactive protein–triglyceride glucose index for the detection of the severity of coronary artery disease in different glucose metabolic states in patients with H-type hypertension
Source: Cardiovasc Diabetol. 2026 Aug 9;25:295. doi: 10.1186/s12933-026-03318-6 (PMC13625319; doi:10.1186/s12933-026-03318-6)
Supplement: Supplementary file 1 [file 12933_2026_3318_MOESM1_ESM.docx]

**Additional file**

**Supplementary figures**

**Fig. S1** SHAP dependence curves for associations between key independent associated variables and coronary lesion risk. (A)Fasting plasma glucose (FPG); (B) Body mass index (BMI); (C) White blood cell count (WBC);(D) Sex; (E) METS‑IR index.

**Fig. S2** Restricted cubic spline curves for the association between CTI and moderate‑to‑severe coronary stenosis after covariate adjustment in hypertensive patients, overall and by glucose metabolic states. (A) Over all; (B) NGR group patients, (C) Pre-DM group patients, (D) DM group patients. OR, odds ratios; CTI C-reactive protein-triglyceride-glucose index; NGR, Normal glucose regulation; Pre-DM, Pre-diabetes mellitus; DM, Diabetes mellitus.

**Fig. S3** To evaluate the joint association of CTI levels and Hcy levels with the risk of CAD severity, we performed multivariable logistic regression analyses, patients were classified into six combined exposure groups according to Hcy levels (<15 μmol/L vs. ≥15 μmol/L) and CTI tertiles (T1, T2, T3), with the group of Hcy <15 μmol/L and CTI T1 serving as the reference. (A): unadjusted; (B): adjusting for sex, age, BMI, SBP, DBP; (C): adjusting for sex, age, BMI, SBP, DBP, smoking, drinking, CCB, statin, antiplatelet agents, beta-blockers, and ACEI/ARB.

CTI C-reactive protein-triglyceride-glucose index; CAD coronary artery disease; OR odds ratio; CI confidence interval; BMI body mass index; SBP systolic blood pressure; DBP diastolic blood pressure

 **Fig. S4** Restricted cubic spline curves for the association between CTI and moderate‑to‑severe coronary stenosis after covariate adjustment in H-type hypertensive patients, overall and by glucose metabolic states. (A) Over all; (B) NGR group patients, (C) Pre-DM group patients, (D) DM group patients. OR, odds ratios; CTI C-reactive protein-triglyceride-glucose index; NGR, Normal glucose regulation; Pre-DM, Pre-diabetes mellitus; DM, Diabetes mellitus.

**Fig. S5** Subgroup analyses of the effect of the CTI on the incidence of moderate‑to‑severe coronary stenosis in hypertension patients. Each subgroup analysis was adjusted for Sex, age, BMI, SBP, DBP, Smoking, Drinking, CCB, Statin, Antiplatelet, Beta blockers and ACEI/ARB.

**Supplementary Tables**

| Table S1 Baseline characteristics according to Gensini score tertiles and degree of coronary stenosis | | | | | | | | | |
| --- | --- | --- | --- | --- | --- | --- | --- | --- | --- |
| characteristics | Tertiles of Gensini score | | | P value | | degree of coronary stenosis | | P value | |
|  | Lowest tertile  ≤12(n=522) | Mid tertile 12-35(n=434) | Highest tertile  ≥35(n=436) | |  | Mild stenosis(n=522) | Moderate-to-severe stenosis(n=870) | |  |
| Sex, n (%) |  |  |  | | ＜0.001 |  |  | | 0.001 |
| Female | 275(52.7%) | 212(48.8%) | 165 (37.8%) | |  | 275(52.7%) | 377(43.3%) | |  |
| Male | 247(47.3%) | 222(51.2%) | 271(62.2%) | |  | 247(47.3%) | 493(56.7%) | |  |
| Age, years | 68.00(61.00, 74.00) | 68.50(61.00, 74.00) | 69.00(61.00, 75.00) | | 0.508 | 68.00(61.00, 74.00) | 69.00(61.00, 75.00) | | 0.307 |
| Height, m | 1.64(1.60, 1.70) | 1.64(1.59, 1.70) | 1.68(1.60, 1.72) | | 0.001 | 1.64(1.60, 1.70) | 1.65(1.60, 1.70) | | 0.042 |
| Weight, kg | 64.00(58.00, 70.00) | 67.45(60.00, 75.00) | 70.00(62.00, 75.00) | | ＜0.001 | 64.00(58.00, 70.00) | 68.00(60.00, 75.00) | | ＜0.001 |
| BMI, kg/㎡ | 23.83(22.15, 25.61) | 24.80(23.38, 27.01) | 24.97(23.20, 26.70) | | ＜0.001 | 23.83(22.15, 25.61) | 24.86(23.30, 26.89) | | ＜0.001 |
| SBP, mmHg | 144.00(132.00, 157.00) | 144.00(132.00, 160.00) | 145.00(130.00, 159.00) | | 0.680 | 144.00(132.00, 157.00) | 144.00(130.00, 160.00) | | 0.675 |
| DBP, mmHg | 84.00(76.00, 91.00) | 84.00(75.75, 93.00) | 82.00(75.00, 91.75) | | 0.467 | 84.00(76.00, 91.00) | 84.00(75.00, 92.00) | | 0.996 |
| Smoking, n (%) | 136(26.1%) | 143(32.9%) | 162(37.2%) | | 0.001 | 136(26.1%) | 305(35.1%) | | ＜0.001 |
| Drinking, n (%) | 50(9.6%) | 38(8.8%) | 46 (10.6%) | | 0.668 | 50(9.6%) | 84(9.7%) | | 0.963 |
| Hyperuricemia, n (%) | 72(13.8%) | 65 (15.0%) | 71(16.3%) | | 0.560 | 72(13.8%) | 136(15.6%) | | 0.351 |
| NAFLD, n (%) | 40(7.7%) | 48(11.1%) | 63(14.4%) | | 0.003 | 40(7.7%) | 111(12.8%) | | 0.003 |
| H-type hypertension, n (%) 283 (54.2%) | | 269(62.0%) | 274 (62.8%) | | 0.010 | 283(54.2%) | 543(62.4%) | | 0.003 |
| Laboratory measurements | | | | | | | | | |
| RBC(×10^12^/L) | 4.44(4.09, 4.76) | 4.48(4.13, 4.86) | 4.47(4.12, 4.87) | | 0.130 | 4.44(4.09, 4.76) | 4.47(4.13, 4.87) | | 0.043 |
| WBC(×10^9^/L) | 5.86 (4.82, 7.00) | 6.19(5.21, 7.40) | 6.66(5.60, 8.23) | | ＜0.001 | 5.86(4.82, 7.01) | 6.32(5.39, 7.75) | | ＜0.001 |
| Homocysteine (μmol/L) 15.47(12.53, 19.20) | | 16.54(13.09, 21.31) | 16.75(13.31, 21.48) | | ＜0.001 | 15.47(12.53, 19.20) | 16.70(13.25, 21.38) | | ＜0.001 |
| Hb (g/L) | 134.00(123.00, 144.25) | 135.00(124.00, 146.00) | 133.00(124.00, 147.00) | | 0.502 | 134.00(123.00, 144.25) | 134.00(124.00, 146.00) | | 0.327 |
| NEUT (×10^9^/L) | 63.10 (57.00, 69.70) | 64.20(58.20, 69.40) | 66.90 (60.15, 74.38) | | ＜0.001 | 63.10(57.00, 69.70) | 65.20(59.00, 71.80) | | ＜0.001 |
| Lymphocyte (×10^9^/L) | 28.10(22.10, 33.93) | 27.55(22.28, 32.63) | 24.40(18.00, 31.18) | | ＜0.001 | 28.10(22.10, 33.93) | 26.40(20.20, 32.03) | | ＜0.001 |
| CRP (mg/L) | 0.78(0.30, 1.80) | 1.02 (0.40, 2.51) | 1.51(0.51, 4.25) | | ＜0.001 | 0.78(0.30, 1.80) | 1.21(0.50, 3.10) | | ＜0.001 |
| Platelet (×10^9^/L) | 209.00(171.00, 246.13) | 211.00(180.00, 250.00) | 214.00(176.03, 255.75) | | 0.180 | 209.00(171.00, 246.13) | 212.00(177.45, 253.00) | | 0.077 |
| FPG (mmol/L) | 5.79(5.05, 6.91) | 6.49(5.39, 8.79) | 7.31(5.78, 10.27) | | ＜0.001 | 5.79(5.05, 6.91) | 6.93(5.56, 9.45) | | ＜0.001 |
| HbA1c (%) | 6.00(5.50, 6.40) | 6.20(5.60, 7.10) | 6.30(5.70, 7.28) | | ＜0.001 | 6.00(5.50, 6.40) | 6.20(5.70, 7.20) | | ＜0.001 |
| TC (mmol/L) | 4.41(3.58, 5.10) | 4.56(3.72, 5.25) | 4.61 (3.76, 5.49) | | 0.012 | 4.41(3.58, 5.10) | 4.58(3.75, 5.36) | | 0.009 |
| TG (mmol/L) | 1.41(0.94, 2.14) | 1.69(1.17, 2.50) | 1.93(1.26, 2.70) | | ＜0.001 | 1.41(0.94, 2.14) | 1.78(1.19, 2.63) | | 0.001 |
| LDL-C (mmol/L) | 2.31(1.77, 2.90) | 2.49(1.83, 3.05) | 2.48(1.95, 3.21) | | 0.001 | 2.31(1.77, 2.90) | 2.49(1.90, 3.14) | | ＜0.001 |
| HDL-C (mmol/L) | 1.27(1.06, 1.53) | 1.18(0.99, 1.38) | 1.15(0.97, 1.38) | | ＜0.001 | 1.27(1.06, 1.53) | 1.16(0.98, 1.38) | | ＜0.001 |
| ALT (U/L) | 19.00(14.00, 27.00) | 20.00(15.00, 29.00) | 21.00(15.00, 31.00) | | 0.075 | 19.00(14.00, 27.00) | 20.00(15.00, 30.00) | | 0.045 |
| AST (U/L) | 21.50(18.00, 26.00) | 21.00(17.00, 27.00) | 21.00(18.00, 30.00) | | 0.385 | 21.50(18.00, 26.00) | 21.00(17.00, 28.00) | | 0.791 |
| UA (μmol/L) | 348.50(286.75, 427.50) | 368.50(306.75, 443.00) | 373.00(317.25, 446.00) | | ＜0.001 | 348.50(286.75, 427.50) | 370.50(314.00, 444.25) | | ＜0.001 |
| Medications, n (%) | | | | |  |  |  | |  |
| CCB | 197(37.7%) | 195(44.9 %) | 180(41.3%) | | 0.079 | 197(37.7%) | 375(43.1%) | | 0.049 |
| Statin | 35(6.7%) | 35(8.1%) | 32(7.3%) | | 0.724 | 35(6.7%) | 67(7.7%) | | 0.490 |
| Antiplatelet | 48(9.2%) | 39(9.0%) | 40(9.2%) | | 0.993 | 48(9.2%) | 79(9.1%) | | 0.943 |
| Beta blockers | 71(13.6%) | 62(14.3%) | 53(12.2%) | | 0.640 | 71(13.6%) | 115(13.2%) | | 0.839 |
| ACEI/ARB | 122(23.4%) | 102(23.5%) | 100(22.9%) | | 0.979 | 122(23.4%) | 202(23.2%) | | 0.948 |
| Glucose metabolism state, n (%) | | | | | ＜0.001 |  |  | | ＜0.001 |
| NGR | 302(57.9%) | 197(45.4%) | 156(35.8%) | |  | 302(57.9%) | 353(40.6%) | |  |
| Pre-DM | 174(33.3%) | 136(31.3%) | 128(29.4%) | |  | 174(33.3%) | 264(30.3%) | |  |
| DM | 46(8.8%) | 101(23.3%) | 152(34.9%) | |  | 46(8.8%) | 253(29.1%) | |  |
| CTI | 8.69(8.16, 9.42) | 9.15(8.54, 9.86) | 9.51(8.92, 10.38) | | ＜0.001 | 8.69(8.16, 9.42) | 9.32(8.73, 10.07) | | ＜0.001 |
| Gensini score | 6.00(4.00, 9.00) | 24.00(18.00, 30.00) | 46.00(39.25, 56.00) | | ＜0.001 | 6.00(4.00, 9.00) | 35.00(24.00, 46.00) | | ＜0.001 |
| TYG index | 8.78(8.35, 8.32) | 9.12(8.66, 9.68) | 9.35(8.89, 9.85) | | ＜0.001 | 8.78(8.35, 9.32) | 9.24(8.77, 9.78) | | ＜0.001 |
| METS-IR index | 21.19(19.13, 23.23) | 22.67(20.56, 24.93) | 22.97(20.62, 25.28) | | ＜0.001 | 21.19(19.13, 23.23) | 22.81(20.58, 25.09) | | 0.001 |
| Number of vessels with stenosis | | | | | ＜0.001 |  |  | | ＜0.001 |
| Single-vessel CAD | 496(95.0%) | 118(27.2%) | 2(0.5%) | |  |  |  | |  |
| Multi-vessel CAD | 26(5.0%) | 316(72.8%) | 434(99.5%) | |  |  |  | |  |
| Body Mass Index (BMI), Systolic Blood Pressure (SBP), Diastolic Blood Pressure (DBP), Non-alcoholic Fatty Liver Disease (NAFLD), Red Blood Cell (RBC), White Blood Cell (WBC), Hemoglobin (Hb), Neutrophil (NEUT), C-reactive Protein (CRP), Fasting Plasma Glucose (FPG), Glycated Hemoglobin A1c (HbA1c), Total Cholesterol (TC), Triglyceride (TG), Low-Density Lipoprotein Cholesterol (LDL-C), High-Density Lipoprotein Cholesterol (HDL-C), Alanine Aminotransferase (ALT), Aspartate Aminotransferase (AST), Uric Acid (UA), Calcium Channel Blocker (CCB), Angiotensin-Converting Enzyme Inhibitor (ACEI), Angiotensin Ⅱ Receptor Blocker (ARB), Normal Glucose Regulation (NGR), Pre-Diabetes Mellitus (Pre-DM), Diabetes Mellitus (DM), C-reactive Protein-Triglyceride-Glucose Index (CTI), Triglyceride-Glucose Index (TyG), Metabolic Score for Insulin Resistance (METS-IR), Coronary Artery Disease (CAD). | | | | | | | | | |

| Table S2 Finally Selected Feature Subset of the LASSO Algorithm | | |
| --- | --- | --- |
| Number | Variable | VIF |
| 1 | Sex | 1.053028 |
| 2 | BMI | 2.848507 |
| 3 | CTI | 1.625809 |
| 4 | WBC | 1.178671 |
| 5 | FPG | 1.540624 |
| 6 | METS-IR | 3.191909 |
| VIF: Variance Inflation Factor | | |

| Table S3 Association between CTI and Moderate-to-severe coronary stenosis in hypertension patients | | | | | | | |  |  |
| --- | --- | --- | --- | --- | --- | --- | --- | --- | --- |
| Variables | Model 1 |  |  | Model 2 |  |  | Model 3 |  | |
|  | OR (95%CI) | P value |  | OR (95%CI) | P value |  | OR (95%CI) | | P value |
| CTI | 1.914(1.691-2.165) | ＜0.001 |  | 1.819(1.605-2.063) | ＜0.001 |  | 1.818(1.603-2.062) | | ＜0.001 |
| CTI T1 | Reference |  |  | Reference |  |  | Reference | |  |
| CTI T2 | 2.473(1.896-3.226) | ＜0.001 |  | 2.365(1.801-3.105) | ＜0.001 |  | 2.379(1.810-3.127) | | ＜0.001 |
| CTI T3 | 4.128(3.112-5.477) | ＜0.001 |  | 3.767(2.824-5.026) | ＜0.001 |  | 3.763(2.819-5.025) | | ＜0.001 |

Model 1: unadjusted

Model 2: adjusted for Sex, age, BMI, SBP, DBP, Smoking and Drinking

Model 3: adjusted for the same variables as model 2, with additional covariates, including CCB, statin, antiplatelet, beta blockers and ACEI/ARB

CTI C-reactive protein-triglyceride-glucose index; CAD Coronary Artery Disease; OR odds ratio; CI confidence interval; BMI body mass index; SBP systolic blood pressure; DBP diastolic blood pressure

Table S4 Multivariable linear regression analysis of CTI with ln(Gensini score + 1)

| Model | CTI |  |  |  |  |
| --- | --- | --- | --- | --- | --- |
|  | B (95% CI) | β | P | Adjusted R**^2^** | VIF |
| Model 1 | 0.286(0.244-0.328) | 0.337 | ＜0.001 | 0.113 | 1.000 |
| Model 2 | 0.265(0.223-0.307) | 0.312 | ＜0.001 | 0.137 | 1.030 |
| Model 3 | 0.263(0.221-0.306) | 0.310 | ＜0.001 | 0.135 | 1.036 |

The dependent variable was ln (Gensini socre + 1). Collinearity diagnostics showed that the variance inflation factor (VIF) for all variables was less than 1.1, indicating no significant multicollinearity. Residual analysis satisfied the assumptions of linearity, normality, and homoscedasticity (Durbin–Watson statistic 2.085–2.092).

Model 1: unadjusted

Model 2: adjusted for Sex, age, BMI, SBP, DBP

Model 3: adjusted for Sex, age, BMI, SBP, DBP, Smoking, Drinking, CCB, statin, antiplatelet, beta blockers and ACEI/ARB

B unstandardized regression coefficient; β standardized regression coefficient; CTI C-reactive protein-triglyceride-glucose index; CI confidence interval; BMI body mass index; SBP systolic blood pressure; DBP diastolic blood pressure

| Table S5 Association between CTI and Moderate-to-severe coronary stenosis in H-type hypertension patients | | | | | | | | | | | |  |
| --- | --- | --- | --- | --- | --- | --- | --- | --- | --- | --- | --- | --- |
| Variables | Model 1 |  | |  | Model 2 |  | |  | Model 3 |  | | |
|  | OR (95%CI) | | P value |  | OR (95%CI) | | P value |  | OR (95%CI) | | P value | |
| CTI | 1.842(1.566-2.166) | | ＜0.001 |  | 1.814(1.537-2.140) | | ＜0.001 |  | 1.806(1.531-2.132) | | ＜0.001 | |
| CTI T1 | Reference | |  |  | Reference | |  |  | Reference | |  | |
| CTI T2 | 2.650(1.843-3.810) | | ＜0.001 |  | 2.655(1.830-3.851) | | ＜0.001 |  | 2.631(1.809-3.827) | | ＜0.001 | |
| CTI T3 | 3.581(2.492-5.147) | | ＜0.001 |  | 3.547(2.447-5.140) | | ＜0.001 |  | 3.525(2.425-5.123) | | ＜0.001 | |

Model 1: unadjusted

Model 2: adjusted for Sex, age, BMI, SBP, DBP, Smoking and Drinking

Model 3: adjusted for the same variables as model 2, with additional covariates, including CCB, statin, antiplatelet, beta blockers and ACEI/ARB

CTI C-reactive protein-triglyceride-glucose index; CAD Coronary Artery Disease; OR odds ratio; CI confidence interval; BMI body mass index; SBP systolic blood pressure; DBP diastolic blood pressure

| **Table S6** Associations between CTI and Moderate-to-severe coronary stenosis according to different glucose metabolism states in hypertension patients | | | | | | | | |  |
| --- | --- | --- | --- | --- | --- | --- | --- | --- | --- |
| Glucose metabolism state | Variables | Model 1 | |  | Model 2 | |  | Model 3 | |
|  |  | OR (95%CI) | P value |  | OR (95%CI) | P value |  | OR (95%CI) | P value |
| NGR | CTI | 1.697(1.406-2.050) | ＜0.001 |  | 1.583(1.305-1.922) | ＜0.001 |  | 1.583(1.304-1.922) | ＜0.001 |
|  | CTI T1 |  |  |  |  |  |  |  |  |
|  | CTI T2 | 2.607(1.827-3.721) | ＜0.001 |  | 2.428(1.683-3.503) | ＜0.001 |  | 2.458(1.700-3.556) | ＜0.001 |
|  | CTI T3 | 2.618(1.686-4.065) | ＜0.001 |  | 2.369(1.505-3.729) | ＜0.001 |  | 2.359(1.498-3.717) | ＜0.001 |
| PRE-DM | CTI | 1.655(1.326-2.065) | ＜0.001 |  | 1.593(1.270-1.999) | ＜0.001 |  | 1.609(1.276-2.028) | ＜0.001 |
|  | CTI T1 |  |  |  |  |  |  |  |  |
|  | CTI T2 | 2.176(1.348-3.513) | 0.001 |  | 2.242(1.365-3.682) | 0.001 |  | 2.285(1.373-3.803) | 0.001 |
|  | CTI T3 | 2.913(1.768-4.801) | ＜0.001 |  | 2.810(1.683-4.691) | ＜0.001 |  | 2.917(1.724-4.935) | ＜0.001 |
| DM | CTI | 1.765(1.244-2.503) | 0.001 |  | 1.713(1.204-2.438) | 0.003 |  | 1.721(1.208-2.451) | 0.003 |
|  | CTI T1 |  |  |  |  |  |  |  |  |
|  | CTI T2 | 1.093(0.400-2.985) | 0.863 |  | 1.027(0.370-2.851) | 0.959 |  | 0.996(0.353-2.805) | 0.994 |
|  | CTI T3 | 2.746(1.043-7.229) | 0.041 |  | 2.560(0.370-2.851) | 0.062 |  | 2.582(0.949-7.029) | 0.396 |
| Model 1: unadjusted  Model 2: adjusted for Sex, age, BMI, SBP, DBP, Smoking and Drinking  Model 3: adjusted for the same variables as model 2, with additional covariates, including CCB, statin, antiplatelet, beta blockers and ACEI/ARB  CTI C-reactive protein-triglyceride-glucose index; CAD Coronary Artery Disease; OR odds ratio; CI confidence interval; BMI body mass index; SBP systolic blood pressure; DBP diastolic blood pressure | | | | | | | | | |

| **Table S7** Associations between CTI and Moderate-to-severe coronary stenosis according to different glucose metabolism states in H-type hypertension patients | | | | | | | | | |
| --- | --- | --- | --- | --- | --- | --- | --- | --- | --- |
| Glucose metabolism state | Variables | Model 1 | |  | Model 2 | |  | Model 3 | |
|  |  | OR (95%CI) | P value |  | OR (95%CI) | P value |  | OR (95%CI) | P value |
| NGR | CTI | 1.651(1.293-2.109) | ＜0.001 |  | 1.598(1.240-2.058) | ＜0.001 |  | 1.601(1.240-2.067) | ＜0.001 |
|  | CTI T1 |  |  |  |  |  |  |  |  |
|  | CTI T2 | 2.855(1.768-4.609) | ＜0.001 |  | 2.768(1.686-4.545) | ＜0.001 |  | 2.860(1.729-4.731) | ＜0.001 |
|  | CTI T3 | 2.192(1.293-3.717) | 0.004 |  | 2.161(1.250-3.736) | 0.003 |  | 2.171(1.250-3.770) | 0.006 |
| PRE-DM | CTI | 1.556(1.152-2.101) | 0.004 |  | 1.570(1.153-2.139) | 0.004 |  | 1.562(1.138-2.143) | 0.006 |
|  | CTI T1 |  |  |  |  |  |  |  |  |
|  | CTI T2 | 2.253(1.152-4.405) | 0.018 |  | 2.242(1.365-3.682) | 0.001 |  | 2.352(1.143-4.839) | 0.020 |
|  | CTI T3 | 2.581(1.313-5.072) | 0.006 |  | 2.413(1.205-4.833) | 0.013 |  | 2.671(1.296-5.504) | 0.008 |
| DM | CTI | 1.822(1.177-2.819) | 0.007 |  | 1.847(1.160-2.940) | 0.010 |  | 1.933(1.190-3.138) | 0.008 |
|  | CTI T1 |  |  |  |  |  |  |  |  |
|  | CTI T2 | 1.091(0.277-4.296) | 0.091 |  | 1.080(0.251-4.653) | 0.918 |  | 1.209(0.264-5.534) | 0.994 |
|  | CTI T3 | 3.081(0.843-11.265) | 0.089 |  | 3.121(0.770-12.654) | 0.111 |  | 3.616(0.854-15.314) | 0.081 |
| Model 1: unadjusted  Model 2: adjusted for Sex, age, BMI, SBP, DBP, Smoking and Drinking  Model 3: adjusted for the same variables as model 2, with additional covariates, including CCB, statin, antiplatelet, beta blockers and ACEI/ARB  CTI C-reactive protein-triglyceride-glucose index; CAD Coronary Artery Disease; OR odds ratio; CI confidence interval; BMI body mass index; SBP systolic blood pressure; DBP diastolic blood pressure | | | | | | | | | |

| **Table S8** The diagnostic value of CTI in assessing the risk of Moderate-to-severe coronary stenosis in different populations | | | | |
| --- | --- | --- | --- | --- |
| Test variables | AUC | SE | 95%CI | P vale |
| ALL | 0.673 | 0.015 | 0.644-0.702 | ＜0.001 |
| NGR | 0.632 | 0.022 | 0.590-0.675 | ＜0.001 |
| Pre-DM | 0.633 | 0.027 | 0.579-0.687 | ＜0.001 |
| DM | 0.650 | 0.042 | 0.567-0.733 | 0.001 |
| H-ALL | 0.661 | 0.020 | 0.621-0.700 | ＜0.001 |
| H-NGR | 0.623 | 0.029 | 0.566-0.680 | ＜0.001 |
| H-Pre-DM | 0.616 | 0.039 | 0.541-0.692 | 0.002 |
| H-DM | 0.666 | 0.053 | 0.562-0.770 | 0.004 |
| NGR: normal glucose regulation group in patients with hypertension; Pre-DM: pre-diabetes group in patients with hypertension; DM: diabetes group in patients with hypertension; H-NGR: normal glucose regulation group in patients with H-type hypertension; H-Pre-DM: pre-diabetes group in patients with H-type hypertension; H-DM: diabetes mellitus group in patients with H-type hypertension; ALL: all patients with hypertension; H-ALL: all patients with H-type hypertension | | | | |

| **Table S9** AUCs of CTI, TYG, FPG and METS-IR index for assessing the severe CAD | | | | |
| --- | --- | --- | --- | --- |
| Variable | AUC | SE | 95%CI | P value |
| Multi-vessel CAD |  |  |  |  |
| All-CTI | 0.711 | 0.014 | 0.683-0.738 | P＜0.001 |
| All-TYG | 0.691 | 0.014 | 0.663-0.718 | P＜0.001 |
| All-FPG | 0.674 | 0.015 | 0.649-0.704 | P＜0.001 |
| All-METS-IR | 0.656 | 0.014 | 0.628-0.685 | P＜0.001 |
| H-CTI | 0.706 | 0.018 | 0.670-0.742 | P＜0.001 |
| H-TYG | 0.683 | 0.019 | 0.646-0.720 | P＜0.001 |
| H-FPG | 0.660 | 0.019 | 0.623-0.670 | P＜0.001 |
| H-MEST-IR | 0.650 | 0.019 | 0.613-0.688 | P＜0.001 |
| Moderate-to-severe stenosis | |  |  |  |
| All-CTI | 0.673 | 0.015 | 0.644-0.702 | P＜0.001 |
| All-TYG | 0.665 | 0.015 | 0.635-0.694 | P＜0.001 |
| All-FPG | 0.660 | 0.015 | 0.631-0.688 | P＜0.001 |
| All-METS-IR | 0.644 | 0.015 | 0.614-0.674 | P＜0.001 |
| H-CTI | 0.661 | 0.020 | 0.621-0.700 | P＜0.001 |
| H-TYG | 0.650 | 0.020 | 0.610-0.690 | P＜0.001 |
| H-FPG | 0.656 | 0.019 | 0.618-0.694 | P＜0.001 |
| H-METS-IR | 0.633 | 0.021 | 0.593-0.674 | P＜0.001 |

Multi-vessel CAD stands for multi-vessel coronary artery disease; CTI for C-reactive protein-triglyceride-glucose index; TyG for triglyceride-glucose index; FPG for fasting plasma glucose; METS-IR for metabolic score for insulin resistance; All-CTI, All -TyG, All -FPG, and All -METS-IR represent CTI, TyG, FPG, and METS-IR in hypertension patients; H-CTI, H-TyG, H-FPG, and H-METS-IR represent CTI, TyG, FPG, and METS-IR in patients with H-type hypertension

| Table S10 Additional prognostic value of CTI and associated measures | | | | | | | |
| --- | --- | --- | --- | --- | --- | --- | --- |
| Population | Model | AUC (95% CI) | P value | NRI (95% CI) | P value | IDI (95% CI) | P value |
| ALL | Basic model | 0.706 (0.679-0.733) |  | Ref |  | Ref |  |
| ALL | +CTI | 0.752 (0.726-0.777) | ＜0.001 | 0.548(0.451-0.648) | ＜0.001 | 0.061(0.049-0.075) | ＜0.001 |
| ALL | +TyG | 0.740 (0.714-0.766) | ＜0.001 | 0.436(0.336-0.538) | ＜0.001 | 0.044(0.033-0.055) | ＜0.001 |
| ALL | +METS-IR | 0.717 (0.690-0.744) | 0.021 | 0.179(0.069-0.290) | ＜0.001 | 0.011(0.005-0.016) | ＜0.001 |
| ALL | +FPG | 0.7334 (0.708-0.760) | ＜0.001 | 0.345(0.248-0.447) | ＜0.001 | 0.033 (0.025-0.043) | ＜0.001 |
| H-type | Basic model | 0.679 (0.642-0.716) |  | Ref |  | Ref |  |
| H-type | +CTI | 0.742 (0.708-0.777) | ＜0.001 | 0.585(0.453-0.726) | ＜0.001 | 0.081(0.062-0.100) | ＜0.001 |
| H-type | +TyG | 0.726 (0.691-0.761) | ＜0.001 | 0.458(0.330-0.594) | ＜0.001 | 0.059(0.043-0.075) | ＜0.001 |
| H-type | +METS-IR | 0.691 (0.655-0.728) | 0.073 | 0.197(0.059-0.338) | 0.006 | 0.011(0.004-0.018) | ＜0.001 |
| H-type | +FPG | 0.718 (0.683-0.753) | ＜0.001 | 0.372(0.243-0.503) | ＜0.001 | 0.042(0.030-0.055) | ＜0.001 |

ALL: all patients with hypertension; H-type: H-type hypertension; CTI for C-reactive protein-triglyceride-glucose index; TyG for triglyceride-glucose index; FPG for fasting plasma glucose; METS-IR for metabolic score for insulin resistance; CI confidence interval; NRI Net Reclassification Improvement; IDI Integrated Discrimination Improvement.

**Table S11** Multiple comparison results of CTI tertiles within each stratification after Benjamini–Hochberg adjustment

| Tables | Subgroup | Comparison | Original P | Benjamini–Hochberg adjusted P | |
| --- | --- | --- | --- | --- | --- |
| Multi-vessel CAD | | | | | |
| Table 3 | All | T2 vs T1 | <0.001 | <0.001 |  |
| Table 3 | All | T3 vs T1 | <0.001 | <0.001 |  |
| Table 4 | Non-H-type | T2 vs T1 | 0.172 | 0.252 |  |
| Table 4 | Non-H-type | T3 vs T1 | 0.023 | 0.033 |  |
| Table 4 | H-type | T2 vs T1 | <0.001 | <0.001 |  |
| Table 4 | H-type | T3 vs T1 | <0.001 | <0.001 |  |
| Table 5 | All-NGR | T2 vs T1 | <0.001 | <0.001 |  |
| Table 5 | All-NGR | T3 vs T1 | <0.001 | <0.001 |  |
| Table 5 | All-Pre-DM | T2 vs T1 | 0.001 | 0.001 |  |
| Table 5 | All-Pre-DM | T3 vs T1 | <0.001 | <0.001 |  |
| Table 5 | All-DM | T2 vs T1 | 0.381 | 0.485 |  |
| Table 5 | All-DM | T3 vs T1 | <0.001 | <0.001 |  |
| Table 6 | H-NGR | T2 vs T1 | <0.001 | <0.001 |  |
| Table 6 | H-NGR | T3 vs T1 | <0.001 | <0.001 |  |
| Table 6 | H-Pre-DM | T2 vs T1 | 0.001 | 0.001 |  |
| Table 6 | H-Pre-DM | T3 vs T1 | <0.001 | <0.001 |  |
| Table 6 | H-DM | T2 vs T1 | 0.527 | 0.599 |  |
| Table 6 | H-DM | T3 vs T1 | 0.006 | 0.012 |  |
| Moderate-to-severe stenosis | | | | |  |
| Table S3 | All | T2 vs T1 | <0.001 | <0.001 |  |
| Table S3 | All | T3 vs T1 | <0.001 | <0.001 |  |
| Table S5 | H-type | T2 vs T1 | <0.001 | <0.001 |  |
| Table S5 | H-type | T3 vs T1 | <0.001 | <0.001 |  |
| Table S6 | All-NGR | T2 vs T1 | <0.001 | <0.001 |  |
| Table S6 | All-NGR | T3 vs T1 | <0.001 | <0.001 |  |
| Table S6 | All-Pre-DM | T2 vs T1 | 0.001 | 0.001 |  |
| Table S6 | All-Pre-DM | T3 vs T1 | <0.001 | <0.001 |  |
| Table S6 | All-DM | T2 vs T1 | 0.994 | 0.994 |  |
| Table S6 | All-DM | T3 vs T1 | 0.396 | 0.485 |  |
| Table S7 | H-NGR | T2 vs T1 | <0.001 | <0.001 |  |
| Table S7 | H-NGR | T3 vs T1 | 0.006 | 0.012 |  |
| Table S7 | H-Pre-DM | T2 vs T1 | 0.020 | 0.040 |  |
| Table S7 | H-Pre-DM | T3 vs T1 | 0.008 | 0.016 |  |
| Table S7 | H-DM | T2 vs T1 | 0.994 | 0.994 |  |
| Table S7 | H-DM | T3 vs T1 | 0.081 | 0.108 |  |

To minimize the risk of false positives due to multiple comparisons, the false discovery rate (FDR) was controlled using the Benjamini–Hochberg procedure. All: all patients with hypertension; H-type H-type hypertension; Non-H-type Non-H-type hypertension; NGR: normal glucose regulation group in patients with hypertension; Pre-DM: pre-diabetes group in patients with hypertension; DM: diabetes group in patients with hypertension.
